# Structural Isomers: Small Change with Big Difference in Anion Storage

**DOI:** 10.1007/s40820-023-01239-7

**Published:** 2023-11-13

**Authors:** Huichao Dai, Yuan Chen, Yueyue Cao, Manli Fu, Linnan Guan, Guoqun Zhang, Lei Gong, Mi Tang, Kun Fan, Chengliang Wang

**Affiliations:** 1 School of Integrated Circuits, Wuhan National Laboratory for Optoelectronics (WNLO), Huazhong University of Science and Technology, Wuhan, 430074 People’s Republic of China; 2 Wenzhou Advanced Manufacturing Institute, Huazhong University of Science and Technology, Wenzhou, 325035 People’s Republic of China; 3 Hubei Key Laboratory of Polymer Materials, School of Materials Science and Engineering, Hubei University, Wuhan, 430062 People’s Republic of China; 4 School of Chemistry and Environmental Engineering, Wuhan Institute of Technology, Wuhan, 430073 People’s Republic of China

**Keywords:** Zinc-organic batteries, Isomers, Solid-state molecular rearrangement, Anion storage, P-type organic electrode materials

## Abstract

Organic electrode materials are promising for batteries. However, the reported organic electrodes are often facing the challenges of low specific capacity, low voltage, poor rate capability and vague charge storage mechanisms, etc. Isomers are good platform to investigate the charge storage mechanisms and enhance the performance of batteries, which, however, have not been focused in batteries. Herein, two isomers are reported for batteries. As a result, the isomer tetrathiafulvalene (TTF) could store two monovalent anions reversibly, deriving an average discharge voltage of 1.05 V and a specific capacity of 220 mAh g^−1^ at a current density of 2 C. On the other hand, the other isomer tetrathianaphthalene could only reversibly store one monovalent anion and upon further oxidation, it would undergo an irreversible solid-state molecular rearrangement to TTF. The molecular rearrangement was confirmed by electrochemical performances, X-ray diffraction patterns, nuclear magnetic resonance spectra, and ^1^H detected heteronuclear multiple bond correlation spectra. These results suggested the small structural change could lead to a big difference in anion storage, and we hope this work will stimulate more attention to the structural design for boosting the performance of organic batteries.

**Supplementary Information:**

The online version contains supplementary material available at 10.1007/s40820-023-01239-7.

## Highlights

- The effect of small changes in isomers on electrochemical performance have not been focused in batteries and two isomers are reported for Zn-ion batteries here.
- The isomer tetrathiafulvalene (TTF) could store two monovalent anions reversibly, showing remarkable performance that outperformed most of the reported organic electrode materials for zinc-ion batteries.
- The isomer tetrathianaphthalene (TTN) could only reversibly store one monovalent anion and upon further oxidation, it would undergo an irreversible solid-state molecular rearrangement to TTF.

**Supplementary Information:**

The online version contains supplementary material available at 10.1007/s40820-023-01239-7.

## Graphical abstract

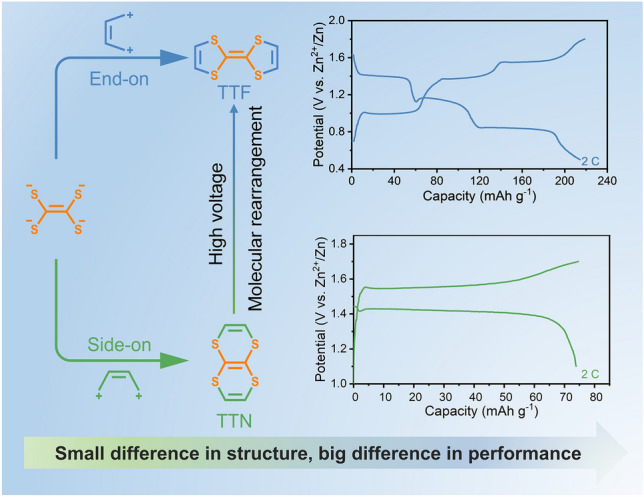

## Introduction

Organic electrode materials have attracted a lot of attention for batteries, due to their flexibility, accessibility from vast natural green resources, recyclability, ease of performance adjustment through molecular design [1–3]. The weak intermolecular interactions which are intrinsic characters of organic materials make it possible to store ions such as Li^+^, Na^+^, K^+^, and anions with high capacity [4–8]. Till now, a lot of organic electrode materials have been reported for batteries [6, 9–11]. However, this emerging field of organic batteries still lacks enough guidelines to lead the further studies and the reported organic electrodes are often facing the challenges of low specific capacity, low voltage, poor rate capability and vague charge storage mechanisms etc.

Isomers, which are ubiquitous in nature, share the same molecular formula but have the different structures, and hence often exhibit distinct physicochemical properties [12–14], attracting great interest in catalysis, food, pharmacy, optics, and other fields [15–19]. Isomers with redox activity and small structural differences are hence attractive for investigating the charge storage mechanisms, which should be able to guide the future studies for enhancing the performance of batteries. However, isomers have not been paid sufficient attention in batteries.

**Fig. 1:**
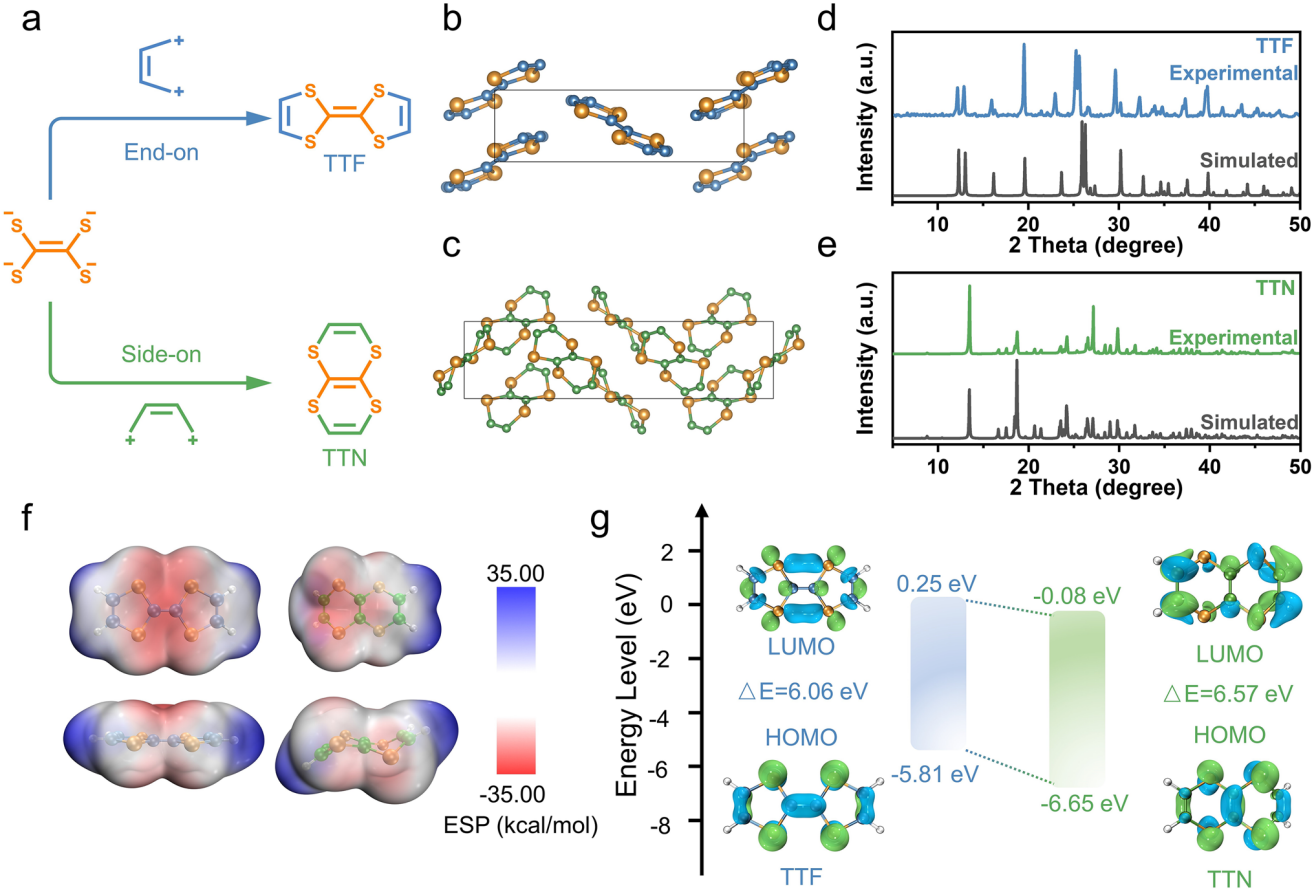
**a** Schematic of two isomers TTF and TTN. **b-e** Perspective view of the unit cell observed from a axis for isomers and comparison of the powders XRD patterns with their respective XRD data simulated based on CIF data (CCDC No. 618630 and 1167371) [22, 31]. **b** Perspective view of TTF. **c** Perspective view of TTN. **d** XRD patterns of TTF. **e** XRD patterns of TTN. **f** Calculated ESP distribution of TTF (left) and TTN (right) molecules. **g** Calculated HOMO/LUMO energy levels and band gap of TTF and TTN

Herein, we reported two p-type isomers, i.e., tetrathiafulvalene (TTF) and tetrathianaphthalene (TTN) for zinc-ion batteries. The p-type organic electrode materials are expected to deliver high-voltage output, with storage of anions [5, 20, 21]. The two isomers were constructed from tetrathiasubstituted ethene by connecting its sulfur centers with ethene bridges in an end-on (TTF) and a side-on (TTN) style, respectively (Fig. 1a) and hence were differed in the bridging ways, containing two five-membered rings for TTF and two six-membered rings for TTN. However, the two isomers exhibited quite different performance in anion storage capabilities. Specifically, TTF could store two monovalent anions reversibly, delivering an average discharge voltage of 1.05 V and a specific capacity of 220 mAh g^−1^ at a current density of 2 C. The discharge voltage and capacity of TTF are remarkable and the specific energy outperformed most of the reported organic electrode materials for zinc-ion batteries. On the other hand, the average discharge voltage of TTN is even higher (at 1.42 V), which, however, could only reversibly store one monovalent anion. Upon further oxidation (> ~ 1.75 V), TTN would undergo an irreversible solid-state molecular rearrangement to TTF. The molecular rearrangement was confirmed by electrochemical performances, X-ray diffraction (XRD) patterns, nuclear magnetic resonance (NMR) spectra, and ^1^H detected heteronuclear multiple bond correlation (HMBC) spectra. In addition, though with storage of more anions, TTF still showed higher rate capability than that of TTN, due to the higher electrical conductivity, higher ion diffusion coefficient and smaller charge transfer resistance of TTF. These results suggested the small structural change could lead to a big difference in anion storage, and we hope this work will stimulate more attention to the isomers and the structural design for boosting the performance of organic batteries.

## Experimental Section

### Materials

All commercially available reagents and solvents were purchased from Energy Chemical, Aladdin or Sinopharm Chemical Reagent Co., Ltd, and used directly without additional purification.

### Preparation of Tetrathianaphthalene

TTN was prepared according to previous report with slight modification [22]. The synthesis of the precursor was detailed in the supporting information (Scheme S1 and see Precursor Preparation in the Supporting Information for details). Under inert gas protection, 3 g of sodium (130 mmol) and 50 mL of absolute ethanol were added to a 1 L three necked flask, and after the sodium was completely dissolved, 165 mL of anhydrous THF was added through a constant pressure dropping funnel. 4,5-bis(benzoylthio)-1,3-dithiole-1-thione (5.3 g, 13 mmol in 80 mL of THF) and cis-1,2-dichloroethylene (2.6 g, 27 mmol in 80 mL of THF) were added dropwise simultaneously over 1 h through the dropping funnel to the refluxing solution. The reaction mixture was refluxed for 12 h. 130 mL of deionized water was added to dissolve the precipitate, then the solvent was removed by rotary evaporation, and the precipitate was collected by filtration and washed with copious amounts of deionized water. The obtained product was further purified by flash column chromatography on silica gel (*i*-hexane) yielding TTN as yellow solid (1.5 g, 56%). ^1^H NMR (600 MHZ, CDCl_3_) δ/ppm 6.45 (s, 4H). ^13^C NMR (600 MHz, CDCl_3_, 300 K) δ/ppm 118.73, 125.43. ESI–MS m/z calculated for [C_6_H_3_S_4_^−^]: 202.91, 203.92, 204.91, 205.91, 206.90; found [C_6_H_3_S_4_^−^]: 202.91, 203.92, 204.91, 205.92.

### Characterizations

XRD were measured by X’Pert3 Powder instrument with Cu Kα radiation. The ^1^H and ^13^C nuclear magnetic resonance (NMR) were conducted on a 600 MHz NMR spectrometer (Bruker, AscendTM 600 MHZ). The ESI/MS was tested by Thermo Fisher Orbitrap LC/MS (Q Exactive). Thermogravimetric analysis (TGA) was performed on a Pyris1 thermogravimetric analyzer by raising the temperature from room temperature to 800 °C at a heating rate of 10 °C min^−1^ under an N_2_ atmosphere. Scanning electron microscopy (SEM) images were acquired from Zeiss field-emission scanning electron microscope. The electrochemical mechanisms were determined by in-situ attenuated total reflection-Fourier transform infrared (ATR-FTIR, Bruker) spectrometer instrument. In-situ ATR-FTIR cell was purchased from the Beijing Scistar Technology Co. Ltd. The GCD tests in the in-situ tests were performed using the Neware battery test system (CT-4008Tn-5V10mA-164, Shenzhen, China). X-ray photoelectron spectroscopy (XPS) data was collected on a Thermo Fisher Lab 250Xi using a monochromic Al X-ray source (1486.6 eV) at Shiyanjia lab (www.Shiyanjia.com). X-band electron paramagnetic resonance (EPR) spectra were recorded on a Bruker EMS nano spectrometer under room temperature.

### Electrochemical Measurements

Rolling method was employed to prepare the electrodes. When conductive additive was Super P, the active material, conductive Super P, and polytetrafluoroethylene (PTFE) binder were mixed in deionized water/isopropanol mixture in a weight ratio of 6:3:1 to form a homogeneous paste, which was then rolled into a thin film and dried at 60 °C in air for 2 h. When the conductive additive was rGO, TTF was dissolved in THF, and then rGO was added at a mass ratio of 5:4. The mixture was ultrasonically dispersed for 12 h, and placed in an oven at 45 °C to slowly volatilize the solvent until the weight did not change. Then PTFE was added at a ratio of 10 wt% and the mixed solvent of deionized water/isopropanol mixture was used as the dispersant. The mixture was rolled into a thin film. The film was punched into small discs with a diameter of 8 mm and an active material mass loading of 2–3 mg cm^−2^, and the free-standing films were pressed onto stainless steel mesh current collectors. The cells were assembled in air by using zinc foil, 3 or 10 m aqueous Zn(ClO_4_)_2_·6H_2_O and glass fiber as counter electrode, electrolyte and separator, respectively. The capacity contribution of rGO or super P was tested by making electrodes of rGO or Super P with PTFE at a ratio of 9:1. The galvanostatic charge/discharge (GCD) tests and galvanostatic intermittent titration technique (GITT) were carried out by using LAND battery test system (Wuhan, China). Cyclic voltammetry (CV) curves and electrochemical impedance spectroscopy (EIS) were performed on a BioLogic VMP3 potentiostat. EIS was collected in the frequency range of 1 MHz to 10 mHz with a voltage amplitude of 10 mV.

### Computational Details

All structures were fully optimized at the level of M062X-D3/def2SVP using the Gaussian 16 program [23–25]. The frequency calculations were performed to confirm the optimized structures being the local minimum on the potential surfaces. The Molecular electrostatic potential (MESP) was carried out by the Multiwfn software [26]. All visualization of MESP and frontier molecular orbitals are performed by the Visual Molecule Dynamics (VMD) software [27]. The density functional theory (DFT) calculations of crystalline TTF and TTN were conducted in Vienna ab initio Simulation package (VASP) with the projector augmented-wave (PAW) pseudopotentials [28, 29]. The Perdew-Burke-Ernzerhof (PBE) generalized-gradient approximation (GGA) functional was adopted [30]. Particularly, the van der Waals (vdW) interaction was described with DFT-D3 method [24]. The Monkhorst–Pack Gamma-centered scheme of k-points sampling for the Brillouin zone was employed with 1 × 1 × 1 for crystalline TTF and TTN. For the band structure calculation, the k-points of them are Γ (0, 0, 0), Z (0, 0.5, 0), D (0, 0.5, 0.5), B (0, 0, 0.5), A (−0.5, 0, 0.5), E (−0.5, 0.5, 0.5), C_2 (−0.5, 0.5, 0) and Y_2 (−0.5, 0, 0).

To distinguish the type of charge transfer kinetics, the *b* values can be fitted by Eq. (1):

1 $$i=a{v}^{b}$$

where the *i* was peak current (mA), and the *v* was scan rate (mV s^−1^). The *b* values could be fitted by the linear relation of log(*i*) = *b*log(*v*) + log(*a*).

The ions diffusion coefficient (*D*) was calculated by galvanostatic intermittent titration technique (GITT) measurement, which follows Eq. (2):

2 $$D = \frac{4}{{\pi \tau }} \times \left( {\frac{{m_B V_M }}{{M_B S}}} \right)^2 \times \left( {\frac{{\Delta E_s }}{{\Delta E_t }}} \right)^2$$

where *τ* was the time of current pulse, *m*_*B*_, *V*_*M*_, *M*_*B*_, and *S* were the mass loading, molar volume, molar mass, and electrode–electrolyte interface area, respectively. *ΔE*_*s*_ was the steady-state voltage change after current pulse, and *ΔE*_*t*_ was the voltage change (V) during the relaxation process.

## Results and Discussion

### Physicochemical Properties of Two Isomers

TTF was purchased commercially, and TTN was synthesized according to previous report with slight modification [22] (Fig. 1a and Scheme S1). All intermediates were characterized to prove their formation and purity by ^13^C NMR (Figs. S1 and S2), and the target product TTN was characterized by ^1^H NMR, ^13^C NMR and HR-MS (Figs. S3–S5). The two isomers were only differed in the bridging ways, containing two five-membered rings for TTF and two six-membered rings for TTN. However, the whole TTF molecule was almost planar (Fig. S6); while, the two six-membered rings of TTN made it non-planar, showing a chair-like conformation (Fig. S7). The two isomers hence showed quite different molecular arrangements (Figs. 1b-c and S8-S9), and the powder X-ray diffraction patterns of them agreed well with the simulated results by using previously reported crystal structures [22, 31] (Figs. 1d-e and S10). In addition, the planar structure of TTF should benefit the formation of larger π-cloud and stronger π-π interactions between molecules, which led to higher thermal stability of TTF than the nonplanar TTN (Fig. S11). Although TTF and TTN had quite similar chemical structures, the NMR spectra of them showed different chemical shifts, due to the different chemical environments. Specifically, the chemical shift of H atoms of the two isomers were 6.31 and 6.45 ppm for TTF and TTN, respectively (Figs. S3 and S12), and in the ^13^C NMR spectra, TTN exhibited two signals located at 125.43 and 118.73 ppm (Fig. S4), while, TTF displayed two chemical shift signals, at 119.10 and 110.14 ppm, respectively (Fig. S13). Moreover, because of the nearly planar structure, TTF exhibited much higher electrical conductivity than that of TTN (Fig. S14 and Table S1).

### Theoretical Calculations of Two Isomers

The electronic states of the two isomers were hence studied by using theoretical calculations. As shown in Fig. 1f, for both of them, the electron cloud was mainly distributed on the sulfur elements with strong electronegativity. However, the electron cloud density on the sulfur elements in TTF was obviously more than that in TTN. This result indicated that the electrons around the sulfur elements in TTF would be easier to lose than that in TTN [32]. In another word, the redox reaction of TTN by losing electrons will have a higher oxidation potential than that of TTF. This point could also be reflected by the highest occupied molecular orbital (HOMO) and valence band minimum (VBM) of these two isomers. As shown in Fig. 1g, the calculated HOMO energy levels of TTF and TTN are -5.81 and -6.65 eV, respectively. The calculated electronic band structure by using the crystal structures indicated that TTN had a lower VBM compared to TTF, which was consistent with the trend of HOMO energy levels of two isomers (Fig. S15). The lower HOMO energy levels and VBM of TTN made it more difficult to lose electrons, resulting in a higher redox potential. In addition, the band gap of TTF was narrower than that of TTN, implying a higher electrical conductivity of TTF, which agreed well with the electrical measurement.

### Redox Activity of Two Isomers

**Fig. 2:**
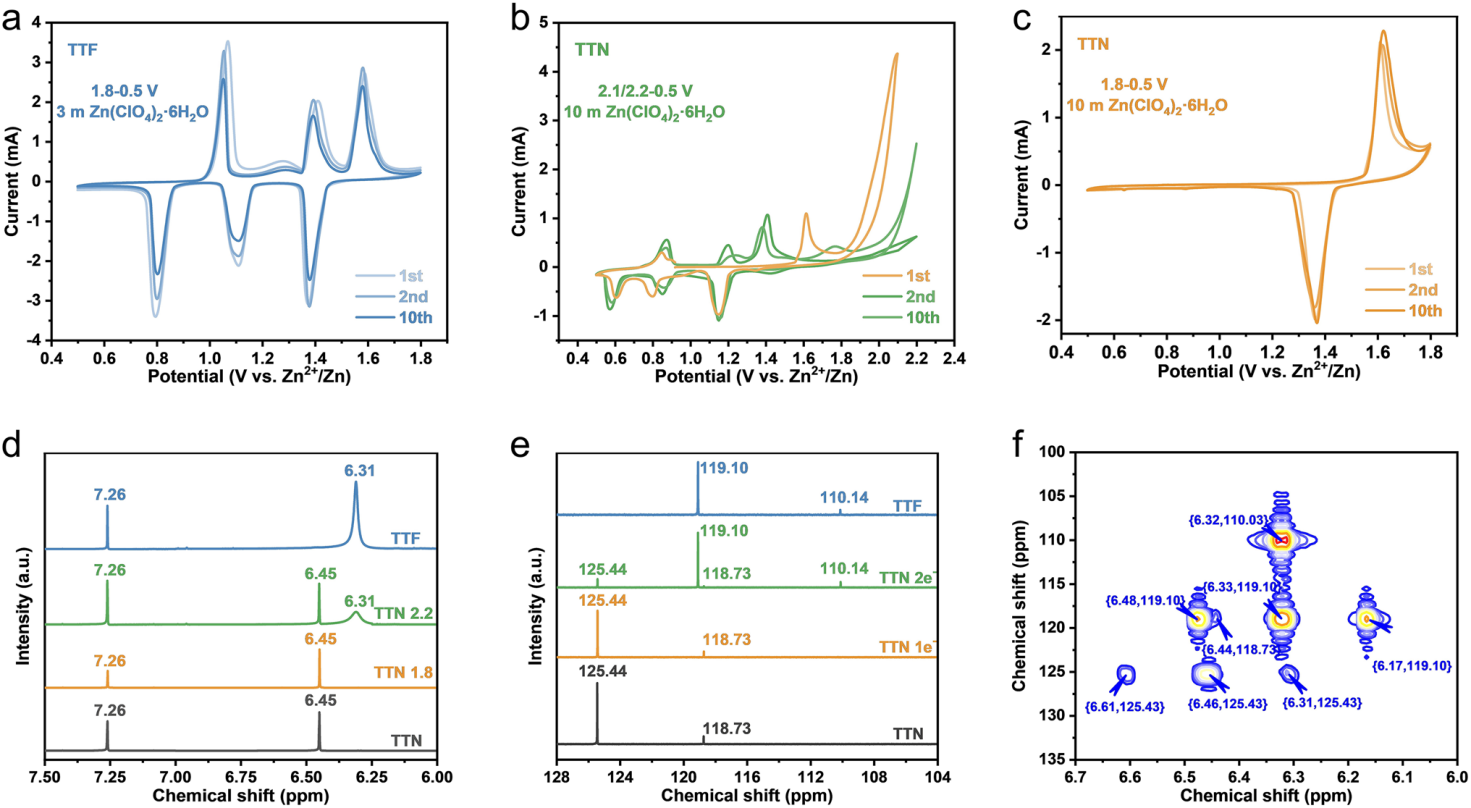
**a** CV curves of TTF electrode at 1 mV s^−1^ in 3 m Zn(ClO_4_)_2_·6H_2_O electrolyte and the voltage range of 0.5–1.8 V. **b** CV curves of TTN electrode at 1 mV s^−1^ in 10 m Zn(ClO_4_)_2_·6H_2_O electrolyte and the voltage range of 0.5–2.1/2.2 V. **c** CV curves of TTN electrode at 1 mV s^−1^ in 10 m Zn(ClO_4_)_2_·6H_2_O electrolyte and the voltage range of 0.5–1.8 V. **d-e** Ex-situ NMR spectrum of TTN, TTN 1.8, TTN 2.2 and TTF electrodes. **d** Ex-situ ^1^H NMR spectra. **e** Ex-situ ^13^C NMR spectra. **f** Ex-situ HMBC spectrum of TTN 2.2 electrode

To evaluate the effect of the change in the structure on the difference in redox ability between these two isomers, cyclic voltammetry (CV) tests were performed on coin cells by using TTF or TTN as cathodes and Zn foil as the counter electrode. Figure 2a showed the typical cyclic voltammetry curve of TTF electrode in 3 m Zn(ClO_4_)_2_·6H_2_O electrolyte at a scan rate of 1 mV s^−1^. Although some other anions, such as TFSI^−^, was considered beneficial to the zinc anode [33–35], we chose Zn(ClO_4_)_2_·6H_2_O as the zinc salt considering the smaller radius and higher solubility of the perchlorate ion for the higher compatibility with the cathode [36, 37]. Three main oxidation peaks at 1.07/1.41/1.59 V (with a shoulder peak at 1.28 V) and three reduction peaks at 1.37/1.11/0.79 V were observed in the first cycle. The CV curves of the following cycles kept similarly with only slight shifts to lower polarization and the shifts might be due to the relaxation of strain/stress after ionic storage [38]. The total electron transfer number calculated by the integrated area of all the peaks was about two for every TTF molecule (Fig. S16). The multiple redox peaks indicated that TTF underwent a reversible phase transition during the ion intercalation/extraction process (Fig. S17). Meanwhile, the current intensity of the subsequent cycles also decreased slightly compared with the initial cycle, which should be ascribed to the slight dissolution of the oxidation state of TTF in aqueous electrolyte (see the following). On the other hand, in the same electrochemical window (0.5 ~ 1.8 V) and same electrolyte (3 m Zn(ClO_4_)_2_·6H_2_O), TTN electrode showed an oxidation peak at 1.63 V in the first cycle and the current continued to rise as the voltage increased (Fig. S18). During the subsequent charging, no oxidation peaks could be observed. Meanwhile, a reduction peak at 1.62 V appeared with the current increasing gradually. Theoretically, every TTN molecule could also lose two electrons, according to the results of TTF and the previous reports on thianthrene-based organic electrode materials [32, 39]. Hence, considering that the electrolyte would not decompose within this voltage range as shown in the TTF-based cells and TTN should have a higher oxidation potential than TTF as mentioned above, the abnormal performance of TTN motivated us to investigate the performance of TTN in higher concentrated electrolyte (10 m Zn(ClO_4_)_2_·6H_2_O). The use of higher concentrated electrolyte for TTN has two reasons: (1) according to the Nernst relation, the redox potential of the p-type TTN electrode would be downshifted due to the increase in the anion activity (see Discussion S1 in the Supporting Information for details) [32] and (2) the higher concentrated electrolyte should have a wider stable electrochemical window for effectively utilizing the redox reaction of TTN [40]. When using higher concentrated electrolyte (10 m Zn(ClO_4_)_2_·6H_2_O) and the cutoff charge voltage was set to 2.1 V, as shown in Fig. 2b, in the first cycle, an obvious oxidation peak appeared at 1.62 V. However, when the voltage was higher than 1.8 V, another oxidation peak started to appear, with dramatically increased current density. Such a current increase at about 1.8 V should not be ascribed to the decomposition of the electrolyte (Fig. S19). When TTN electrode was scanned back, three reduction peaks at 1.15, 0.79 and 0.61 V appeared. After several cycles, the initial oxidation peak at 1.62 V disappeared completely, which was replaced by three pairs of redox peaks at 1.41/1.15, 1.21/0.85, and 0.87/0.57 V. The shape of the cyclic voltammetry curves of the TTN electrode after stabilization was very similar to those of the TTF electrode, prompting us to perform the same test on the TTF electrode in the same highly concentrated electrolyte. Surprisingly, the cyclic voltammetry curves of TTN electrode after stabilization were basically consistent with those of TTF electrode in the same electrolyte (Fig. S20). These results indicated that the TTN electrode probably underwent a solid-state molecular rearrangement to TTF after scanned to high voltages. We therefore tested the TTN electrode in the voltage range of 0.5–1.8 V in the highly concentrated electrolyte, which showed that the oxidation peak at 1.62 V kept stable in subsequent cycles with a stable reduction peak at 1.36 V (Fig. 2c). The total electron transfer number calculated by the integrated area of this peak was about one for every TTN molecule (Fig. S21). These results indicated the further oxidation of TTN was key to the potential solid-state molecular rearrangement to its isomer TTF.

### Solid-State Molecular Rearrangement from TTN to TTF

To confirm the solid-state molecular rearrangement from TTN to its isomer TTF at high voltages, we extracted the discharged states of TTN electrodes, for which the cutoff charged voltage was set to 2.2 V (defined as TTN 2.2) or 1.8 V (defined as TTN 1.8), respectively, by deuterated chloroform. Figure 2d showed the chemical shift information of H atoms in different discharged states, which were compared with those of TTF and TTN. It could be found that when the TTN electrode was only charged to 1.8 V, the chemical environment of its H atoms after discharging was still consistent with the raw material, indicating the TTN was recovered; however, when the TTN electrode was charged to 2.2 V, a new signal at 6.31 ppm was found in ^1^H NMR spectrum after discharging to 0.5 V. The new peak was coincident well with the chemical shift of H atoms in TTF molecule. The ^13^C NMR spectra showed similar phenomena (Fig. 2e). These results confirmed the solid-state molecular rearrangement from TTN to its isomer TTF and implied that the solid-state molecular rearrangement occurred under higher voltage (or further oxidation). ^1^H detected heteronuclear multiple bond correlation (HMBC) spectrum could associate ^1^H nuclei with coupled ^13^C nuclei, providing more evidences. As shown in Fig. 2f, eight sets of correlated signals were observed in HMBC spectra for the TTN electrode that had been charged to 2.2 V and then discharged to 0.5 V. Among them, the two pairs of symmetrically appeared signals were the rotating sideband peaks of TTF ({6.48, 119.10}; {6.17,119.10}) and TTN ({6.61, 125.43}; {6.31, 125.43}) respectively, and their mean rotating sideband peaks was the coupling between hydrogen and its directly connected carbon (TTF {6.33, 119.10} and TTN {6.46, 125.43}). Furthermore, the signals of {6.32, 110.03} and {6.44, 118.73} corresponded to the long-range hydrogen-carbon coupling in TTF and TTN respectively, showing the coupling between hydrogen and the indirectly connected carbon of tetrathiasubstituted ethene. These results could be confirmed by the comparison with pure TTF and TTN (Figs. S22 and S23). Such solid-state molecular rearrangement could be further confirmed by XRD patterns. As shown in Fig. S24, when the TTN electrode was only charged to 1.8 V, the XRD signals after discharging did not change, implying the reversibility of TTN in this voltage range; however, when the TTN electrode was charged to 2.2 V, strong diffraction signals belonging to TTF were observed (mark with *).

All of these results further proved that TTN did undergo a solid-state molecular rearrangement to TTF under high voltage or (further oxidation), and the conversion rate of TTN to TTF was as high as 86% only after one cycle (Fig. S25). Once TTN was converted into TTF, the electrodes showed the similar electrochemical performance with pure TTF electrodes (Figs. S20 and S26). One of the previous reports found that TTN may be converted into TTF when meeting strong base and it proposed a process of deprotonation due to the base that led to the isomerization (Scheme S2) [41]. In this case, fused compounds such as [1, 4] benzodithiino[2,3-b][1,4]benzodithiin (DBTTN) without hydrogen atoms at the ethene bridges, could prevent the molecular rearrangement. However, we found that DBTTN would also experience the similar isomerization under high voltage, suggesting a different isomerization process that was probably more reasonable (Scheme S3, Fig. S27, see Discussion S2 in the Supporting Information for details).

### Electrochemical Performance of Two Isomers

**Fig. 3:**
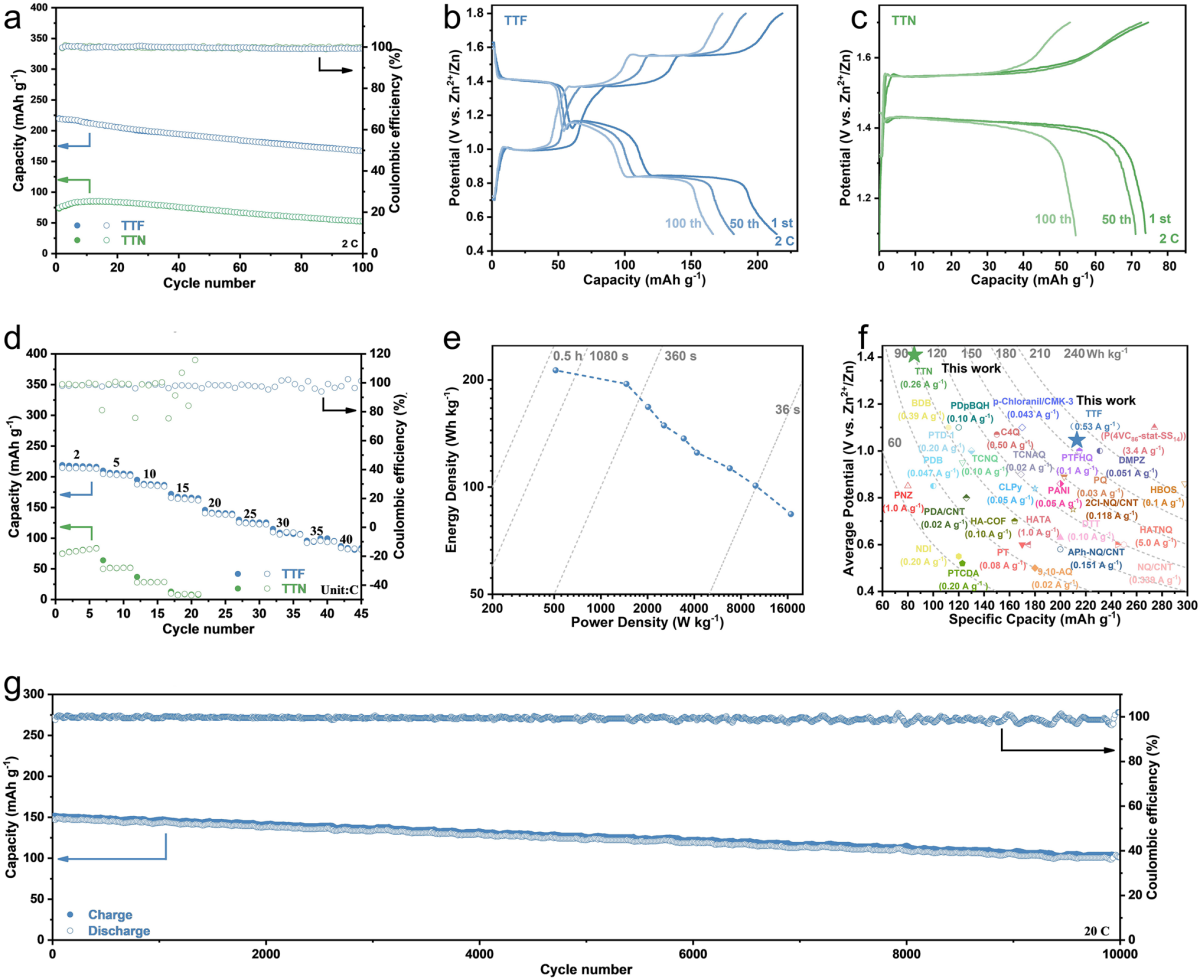
**a** Cycling performance of TTF and TTN electrode at 2 C. **b** Charge–discharge profile of TTF electrode at 2 C. **c** Charge–discharge profile of TTN electrode at 2 C. **d** Rate capability of the TTF and TTN electrode. **e** Ragone plot of TTF//Zn battery. **f** Comparison of TTF and TTN electrode with representative reported organic electrodes including both small molecules and polymers. **g** Cycling stability of TTF electrode at 20 C

Since TTN would undergo a rearrangement reaction to TTF when charged to a higher voltage, we controlled the voltage ranges of TTN and TTF electrode tests at 1.1–1.7 and 0.5–1.8 V, respectively. In this case, TTN and TTF would involve one and two electron transfer, respectively. Thereinto, the loss of two electrons of TTF accompanying with the storage of two monovalent anions resulted into the dissolution of active materials and rapid capacity degradation (Figs. S28 and S29). Hence, for TTF, the conductive additive (Super P) was replaced by reduced graphene oxide (Figs. S29 and S30), which had a stronger interaction with organic electrode materials [42, 43], to improve the cycling stability of TTF electrodes. As a result, the TTF electrodes exhibited a discharge specific capacity of 220 mAh g^−1^ at 2 C with a capacity utilization of 84% (2 electron transfer per TTF molecule) and a capacity retention of 76% after 100 cycles (Fig. 3a). It should be noted that the capacity contribution of reduced graphene oxide was negligible (Fig. S31). On the other hand, within a given voltage range, the initial discharge specific capacity of TTN electrodes was only 75 mAh g^−1^ at 2 C, and the capacity could reach 85 mAh g^−1^ after a short activation, which was corresponding to only 0.65 electron transfer. At a slow rate, the TTN electrodes exhibited a specific capacity of 110 mAh g^−1^ at 0.5 C (Fig. S32 and S33), which was close to the theoretical capacity of 1 electron transfer (131 mAh g^−1^). These results also indicated that TTN showed inferior rate capability compared with TTF. Besides, the average discharge voltage of TTF and TTN electrodes was 1.05 and 1.42 V, respectively (Fig. 3b, c), which were consistent with the peaks of their CV curves. The high capacity as well as the high discharge voltage of TTF therefore led to a high specific energy (213 Wh kg^−1^), which outperformed most of the reported organic electrode materials for Zn-ion batteries (Fig. 3f and Table S2). The rate performance of these two isomers was also investigated. Compared with TTN, TTF electrode exhibited excellent rate performance. As shown in Fig. 3d, the TTF electrode delivered discharge capacities of 215, 203, 186, 164,140, 124, 102, 93, and 81 mAh g^−1^ at the current densities of 2, 5, 10, 15, 20, 25, 30, 35, and 40 C, respectively. In sharp contrast, the TTN electrode exhibited very poor rate performance, yielding only a discharge capacity of 8 mAh g^−1^ at a current density of 15 C. The specific energy of TTF electrodes hence could reach 213, 195,168, 149, 137, 125, 113, 101, and 84 Wh kg^−1^ (based on the electrode materials) at power densities of 511, 1462, 2016, 2554, 3425, 4166, 6780, 9934, and 16,800 W kg^−1^, respectively (Fig. 3e). In addition, the long-term cyclability of TTF electrode was also investigated. As shown in Fig. 3g, at a current density of 20 C, the specific discharge capacity of TTF electrode was still as high as 104 mAh g^−1^ even after 10,000 cycles, corresponding to a capacity retention of 68.8%.

### Charge Storage Mechanism of Two Isomers

**Fig. 4:**
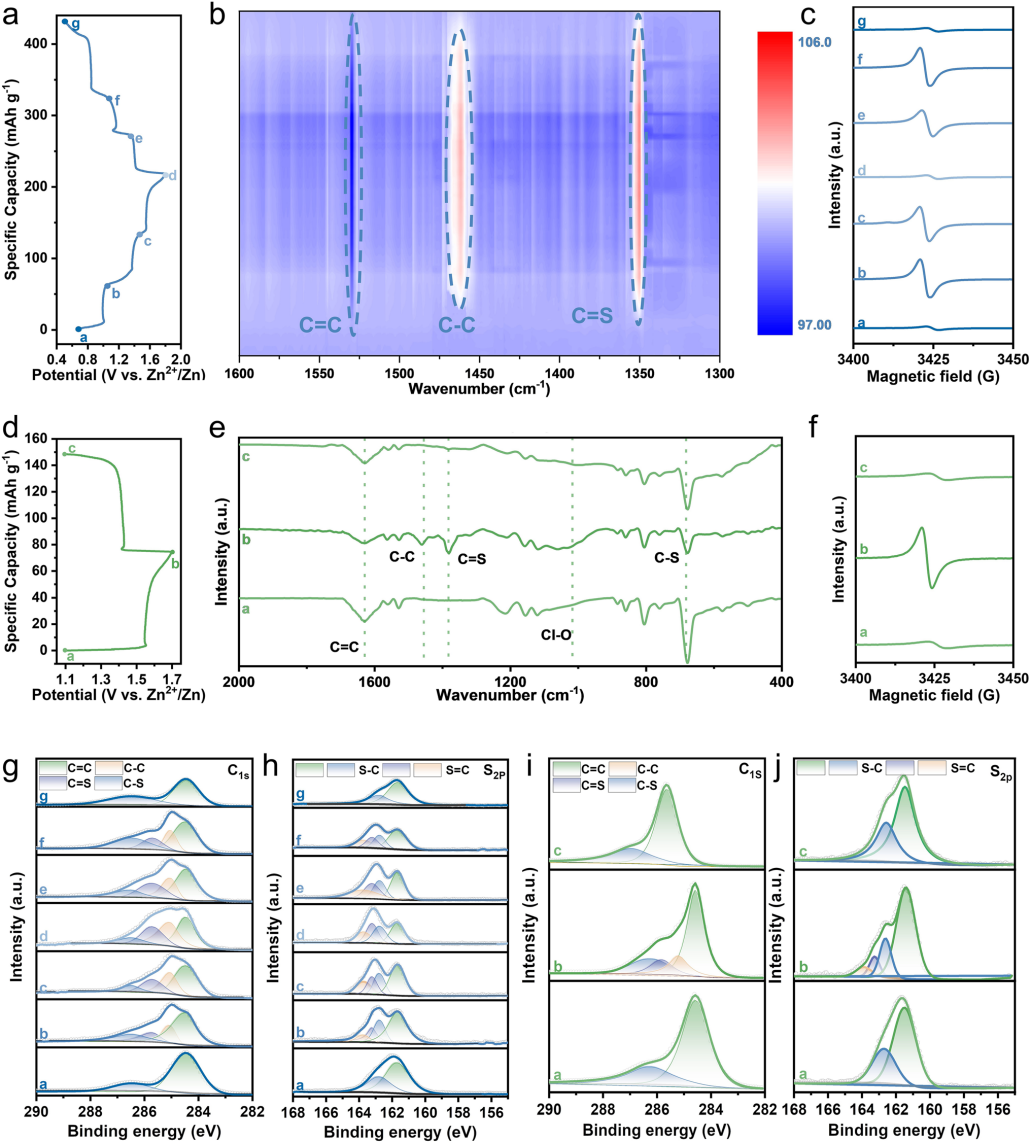
Initial discharge–charge curve of the isomer//Zn battery at 2 C, and the ex-situ tests were conducted at the marked points: **a** TTF//Zn battery; **d** TTN//Zn battery. FTIR tests of these isomer electrodes during cycling: **b** In-situ ATR-FTIR test of TTF electrode; **e** ex-situ FTIR test of TTN electrode. Ex-situ EPR spectra of isomer electrodes in different states: **c** TTF electrode; **f** TTN electrode (the g values of all the EPR spectra were about 2.003). XPS spectra of isomer electrodes in different states: **g** C 1*s* XPS spectra of TTF electrode; **h** S 2*p* XPS spectra of TTF electrode; **i** C 1*s* XPS spectra of TTN electrode; **j** S 2*p* XPS spectra of TTN electrode

To reveal the charge storage mechanism of the two isomers, the electrodes with different discharged and charged states were tested (Fig. 4a, d). As shown in Fig. 4b, e, after charging, the intensity of the peak belonging to C =C bonds in FTIR spectra weakened, accompanying with enhancement of the peak intensity at about 1463 and 1350 cm^−1^ that could be assigned to C–C bonds and C=S bonds, respectively [44–46]. In addition, the weakening of the C-S bond at 679 cm^−1^ of TTN electrode and the absorption at 1015 cm^−1^ attributed to ClO_4_^−^ could be observed in the ex-situ FTIR spectra (Fig. 4e), implying the electron redistribution after oxidation and the storage of ClO_4_^−^ anions [39, 44]. The relative intensities of these peaks showed a reverse tendency during discharging, suggesting the reversibility of both TTF and TTN. High-resolution XPS spectra further confirmed the charge storage mechanism of the two isomers. For TTF electrode, two peaks with binding energy of ~ 285.1 and ~ 285.7 eV became obvious in the C 1*s* spectra after charging, which belonged to C–C and C=S bonds (Fig. 4g) [47]. Similar phenomena could be found in the S 2*p* spectra (Fig. 4h) [48]. The TTN electrode showed similar reversible changes, that is, the binding energy signals belonging to C–C and C=S were enhanced in its oxidation states, and these signals weakened again after discharging (Fig. 4i, j). In addition, signals of Cl 2*p* were detected after charging for both TTF and TTN (Fig. S34). Notably, these changes were restored when the two isomer electrodes were discharged to a fully reduced state. All of these results confirmed that the loss of electrons at the S atom accompanying with the storage of anions, contributed to the capacity, which was agreement well with the previous literatures on similar thio-materials (Scheme S4) [49–52]. More important is that the EPR spectra confirmed the two electron transfer for TTF and one electron transfer for TTN. Strong EPR signal was detected in the intermediate process of TTF, but much weak signal was observed after being fully charged or discharged (Fig. 4c), indicating an even number electron transfer. On the other hand, EPR signal of TTN after being fully charged could be clearly detected (Fig. 4f), suggesting an odd number electron transfer.

### Reaction Kinetics of Two Isomers

The reaction kinetics of them was studied to determine the reason for the difference in rate performance. The CV curves conducted at different scan rates indicated that both isomers exhibited diffusion-controlled behavior [53] (Fig. S35), although the *b* values for TTF electrodes were slightly higher than that of TTN, implying redox reactions of TTF electrode were more accessible than that of TTN electrode. On the other hand, the electrochemical impedance spectroscopy (EIS) showed that the charge transfer resistance of TTF electrode was much smaller than that of the TTN electrode (Fig. S36), which could be ascribed to the much higher electrical conductivity of TTF than TTN as mentioned above. Additionally, galvanostatic intermittent titration (GITT) measurements showed that the anion diffusion coefficient of TTF electrode (about 10^–8^ ~ 10^–9^ cm^2^ s^−1^) was three orders of magnitude higher than that of TTN electrode (Fig. S37). Of special note was that the anion diffusion coefficient of TTF electrode was higher than that of most organic electrode [32, 39, 54–56]. The high electrical conductivity, low charge transfer resistance, and high anion diffusion coefficient of TTF contributed to the high rate performance of TTF electrode.

## Conclusions

In summary, the study of isomers on the electrochemical performance is rarely reported and here we reported two isomers, TTF and TTN, for zinc-ion batteries. The two isomers exhibited quite different performance in anion storage capabilities. Specifically, TTF could store two monovalent anions reversibly, deriving an average discharge voltage of 1.05 V and a specific capacity of 220 mAh g^−1^ at a current density of 2 C. The discharge voltage and capacity of TTF are remarkable and the specific energy outperformed most of the reported organic electrode materials for zinc-ion batteries. On the other hand, the average discharge voltage of TTN is even higher (at 1.42 V), which, however, could only reversibly store one monovalent anion. Upon further oxidation (> ~ 1.75 V), TTN would undergo an irreversible solid-state molecular rearrangement to TTF. In addition, TTF showed higher rate capability than that of TTN, due to the higher electrical conductivity, higher ion diffusion coefficient and smaller charge transfer resistance of TTF. These results suggested the small structural change could lead to a big difference in anion storage. There are a lot of isomers including not only the structural isomers but also the conformational isomers, stereoisomers, geometric isomers and enantiomers etc. We hope this work will stimulate more attention to the isomers and delicate structural design for boosting the performance of organic batteries.

## Supplementary Information

Below is the link to the electronic supplementary material.

Supplementary file1 (PDF 3283 kb)
