# Supplementary material for: Structural Isomers: Small Change with Big Difference in Anion Storage
Source: Nanomicro Lett. 2023 Nov 13;16:13. doi: 10.1007/s40820-023-01239-7 (PMC10643786; doi:10.1007/s40820-023-01239-7)
Supplement: Supplementary file 1 — Supplementary file1 (PDF 3283 kb) [file 40820_2023_1239_MOESM1_ESM.pdf]

Supporting Information for

# Structural Isomers: Small Change with Big Difference in Anion Storage

Huichao Dai[1, †], Yuan Chen[1, 2, †], Yueyue Cao[1], Manli Fu[1], Linnan Guan[1], Guoqun Zhang[1], Lei Gong[1], Mi Tang[3], Kun Fan[4], Chengliang Wang[1, 2, *]

[1] School of Integrated Circuits, Wuhan National Laboratory for Optoelectronics (WNLO), Huazhong University of Science and Technology, Wuhan 430074, P. R. China

[2] Wenzhou Advanced Manufacturing Institute, Huazhong University of Science and Technology, Wenzhou 325035, P. R. China

[3] Hubei Key Laboratory of Polymer Materials, School of Materials Science and Engineering, Hubei University, Wuhan 430062, P. R. China

[4] School of Chemistry and Environmental Engineering, Wuhan Institute of Technology, Wuhan 430073, P. R. China

[†]Huichao Dai and Yuan Chen contributed equally to this work.

*Corresponding author. E-mail: clwang@hust.edu.cn (Chengliang Wang)

## S1 Precursor Preparation

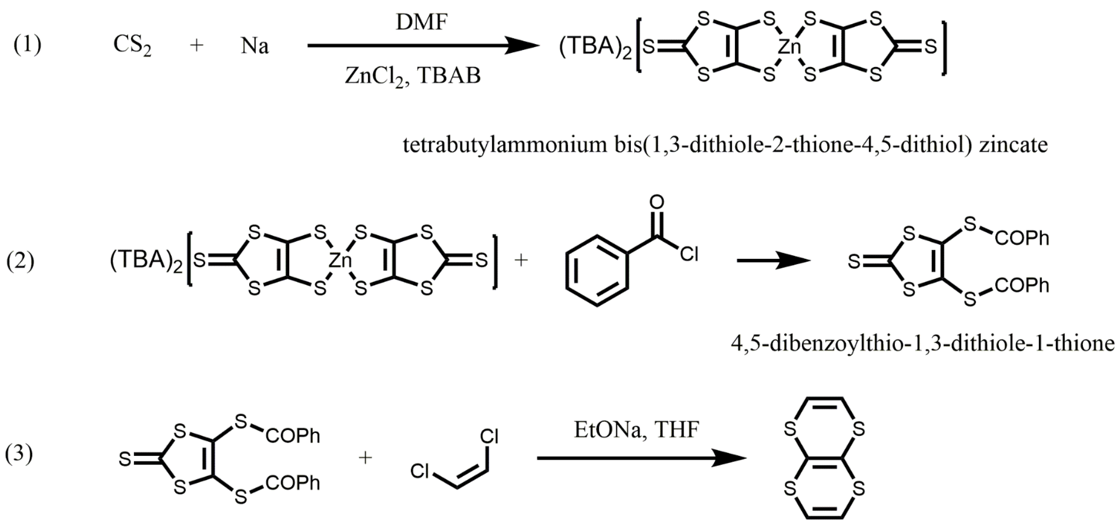


**Scheme S1** Synthetic process of TTN

## S2 Preparation of Tetrabutylammonium Bis(1,3-dithiole-2-thione-4,5-dithiol) Zincate

tetrabutylammonium bis(1,3-dithiole-2-thione-4,5-dithiol) zincate was prepared according to previous report with slight modification [S1]. Under inert gas protection, a 1 L three necked flask was charged with 4.6 g of sodium (200 mmol) and placed in an ice-water bath. Then, 36 mL of carbon disulfide (600 mmol) and 60 mL of dimethylformamide were slowly added dropwise over 45 min. After the addition, the reaction mixture was allowed to warm to 25 °C and stir 12 h, during which time it becomes increasingly deep red/violet. To remove the unreacted sodium, slowly drop 20 mL of methanol in an ice-water bath. Afterwards, added the mixed solution of 80 mL methanol and 100 mL deionized water into the three necked flask quickly. A solution of 4.1 g of $ZnCl_2$ (30 mmol) in 150 mL of concentrated aqueous ammonium hydroxide and 100 mL of methanol was slowly added dropwise to the reaction mixture with vigorous stirring. A solution of tetrabutylammonium bromide (10.5g, 50 mmol) in deionized water (50 mL) was added dropwise at least 1 h, and the solution was stirred overnight. The precipitate was collected by filtration, washed with deionized water, isopropanol and ether, and dried in vacuum to obtain 14 g of red powder in 78% yield. $^{13}C$ NMR (600 MHz, $CDCl_3$, 300 K) δ/ppm 208.86, 136.18, 58.98, 24.13, 19.86, 13.80.

## S3 Preparation of 4,5-dibenzoylthio-1,3-dithiole-1-thione

According to the literature [S1], in a 1 L round-bottomed flask, 16 g of tetrabutylammonium bis(1,3-dithiole-2-thione-4,5-dithio) zincate was dissolved in 400 mL acetone. Then, 40 mL of benzoyl chloride was added via a dropping funnel over 4 h. After stirring for 12 h, the resulting yellow-light brown precipitate was filtered and

washed with water and acetone. These crude products were air-dried and dissolved in 350 mL of chloroform. Afterwards, 0.5 g activated carbon was added and stirred for 10 min under reflux. The mixture was filtered while hot, and the filter cake was washed with 50 mL of hot chloroform. The combined chloroform solution was concentrated to 150 mL, and then 50 mL of methanol was added dropwise at 60°C. The solution was left in the refrigerator overnight to recrystallize and the resulting precipitate was collected and air-dried, affording yellow crystals (8.1 g, 41%). $^{13}C$ NMR (600 MHz, $CDCl_3$, 300 K) δ/ppm 212.36, 185.47, 134.90, 134.82, 133.68, 129.15, 128.00.

## S4 Supplementary Figures and Tables

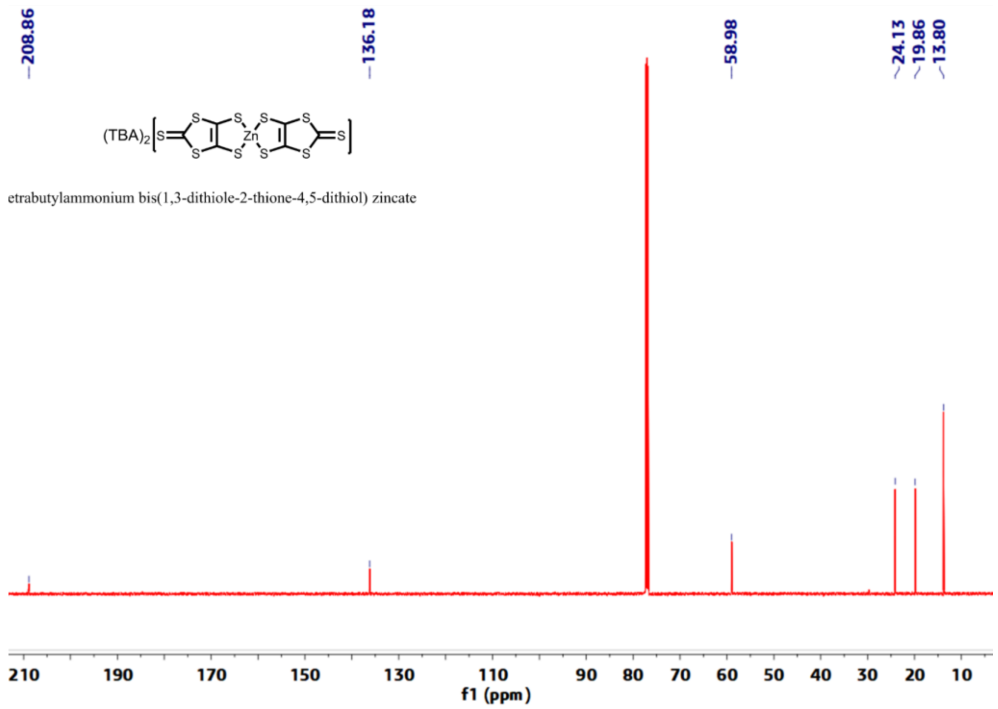


**Fig. S1** $^{13}C$ NMR (600 MHz, $CDCl_3$, 300 K) spectra of tetrabutylammonium bis(1,3-dithiole-2-thione-4,5-dithiol) zincate

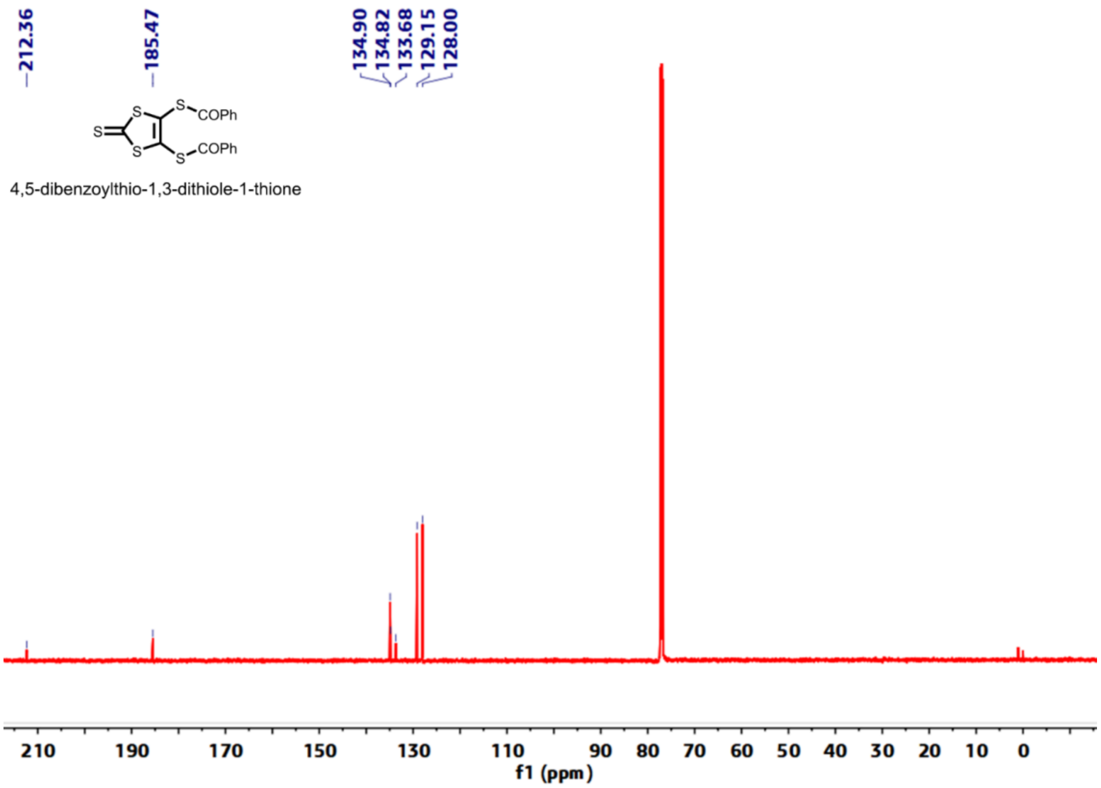


**Fig. S2** $^{13}C$ NMR (600 MHz, $CDCl_3$, 300 K) spectra of 4,5-dibenzoylthio-1,3-dithiole-1-thione

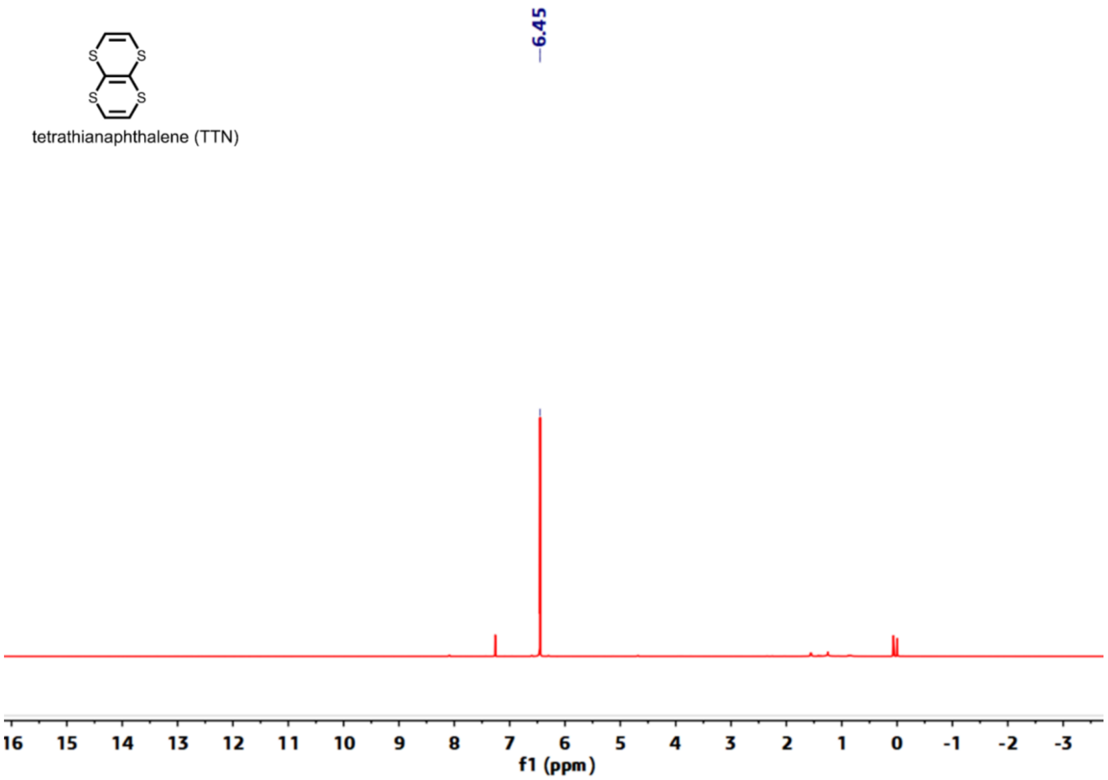


**Fig. S3** $^{1}H$ NMR (600 MHz, $CDCl_3$, 300 K) spectra of TTN

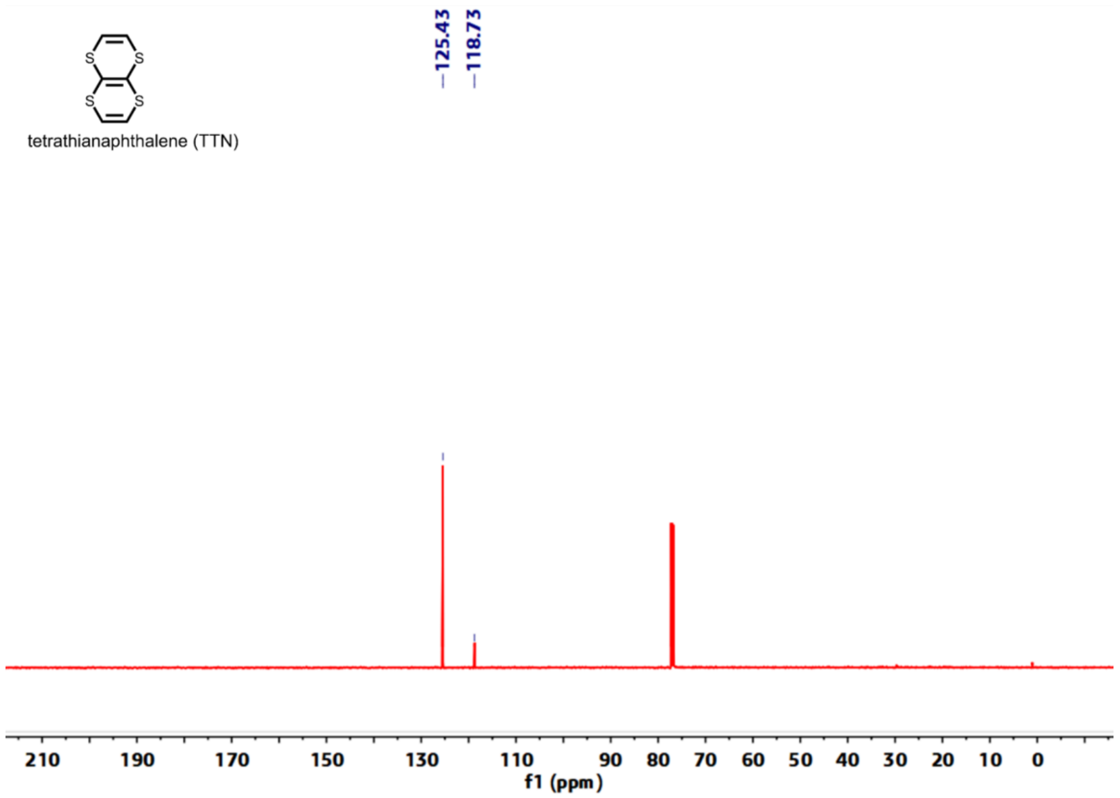


**Fig. S4** $^{13}$C NMR (600 MHz, $CDCl_3$, 300 K) spectra of TTN

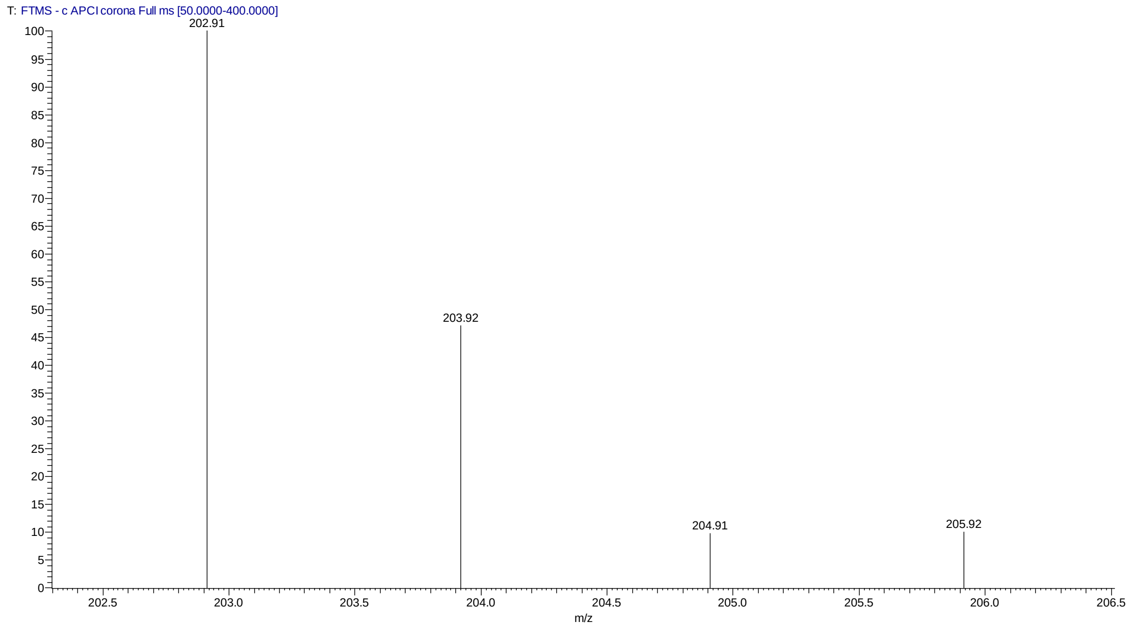


**Fig. S5** ES-/MS date of TTN (negative, DCM as solvent). The peaks were 202.91, 203.92, 204.91, 205.52, which attributed to molecular fragments of $C_6H_3S_4^-$ and its isotopes

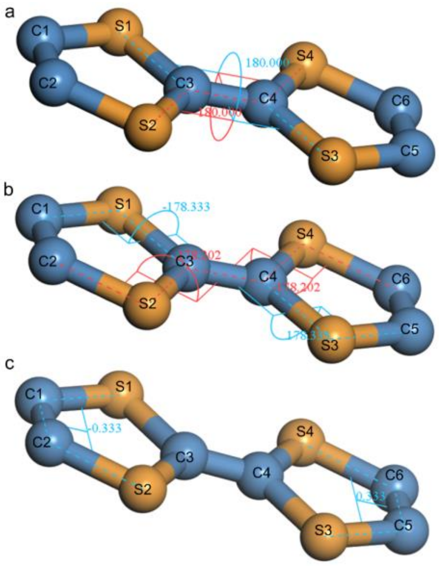


**Fig. S6** The torsion between four atoms in a TTF molecule, showing almost planar structure. **a** S1, C3, C4 and S3 (blue line); S2, C3, C4 and S4 (red line). **b** C1, S1, C3, C4 or C5, S3, C4, C3 (blue line); C2, S2, C3, C4 or C5, S4, C4, C3 (red line). **c** S1, C1, C2, S2 or S4, C6, C5, S3

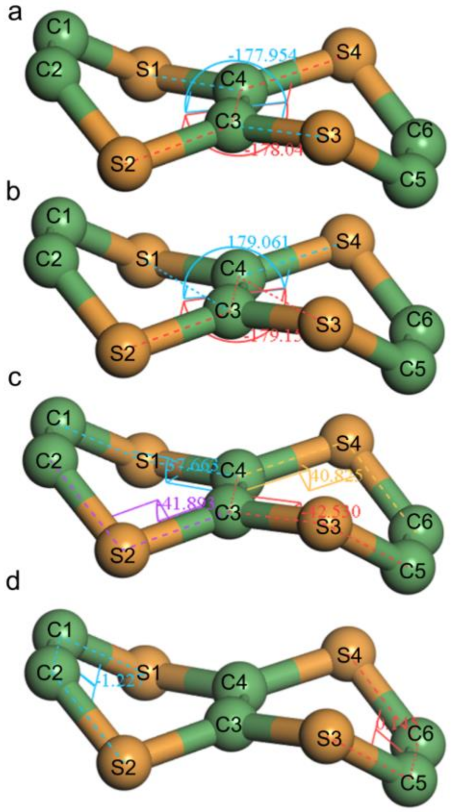


**Fig. S7** The torsion between four atoms in a TTN molecular, showing a non-planar chair-like conformation. **a** S1, C4, C3 and S3 (blue line); S2, C3, C4 and S4 (red line). **b** S1, C3, C4 and S4 (blue line); S2, C3, C4 and S3 (red line). **c** C1, S1, C4 and C3 (blue line); C5, S3, C3, C4 (red line); C2, S2, C3, C4 (purple line); C6, S4, C4, C3 (orange line). **d** S1, C1, C2, S2 (blue line); S4, C6, C5, S3 (red line)

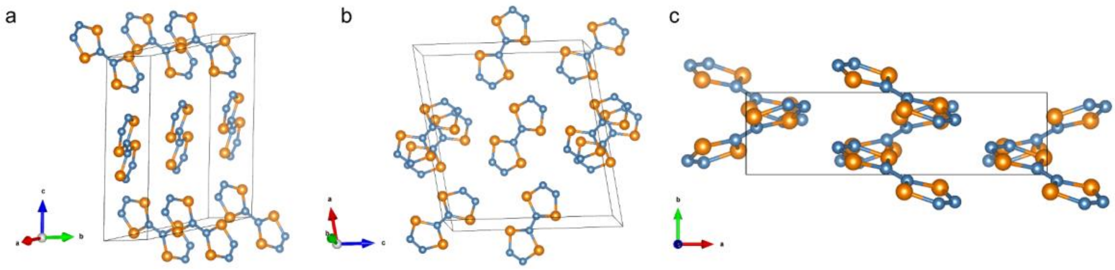


**Fig. S8 a** Perspective view of a TTF unit cell observed from a axis. **b** Perspective view of a TTF unit cell observed from b axis. **c** Perspective view of a TTF unit cell observed from c axis

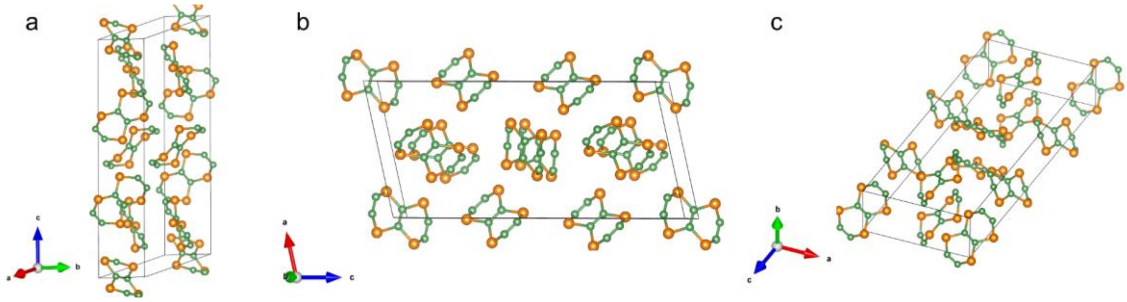


**Fig. S9 a** Perspective view of a TTN unit cell observed from a axis. **b** Perspective view of a TTN unit cell observed from b axis. **c** Perspective view of a TTN unit cell observed from offset c axis

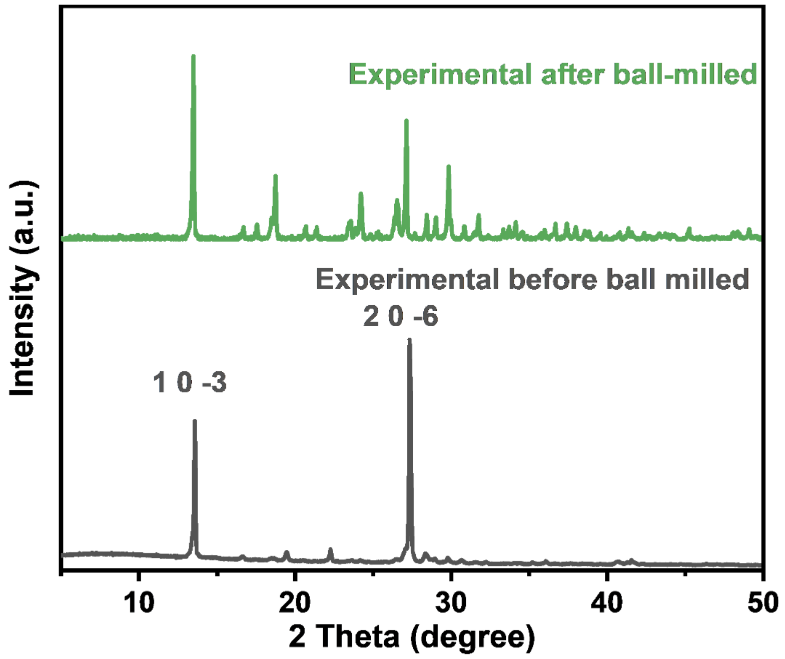


**Fig. S10** Comparison of XRD spectra of TTN before and after ball milled. It should be noted that the obtained TTN grains had a preferred orientation. The powders after ball-milling showed XRD patterns matching well with its theoretical predicted X-ray diffraction peaks

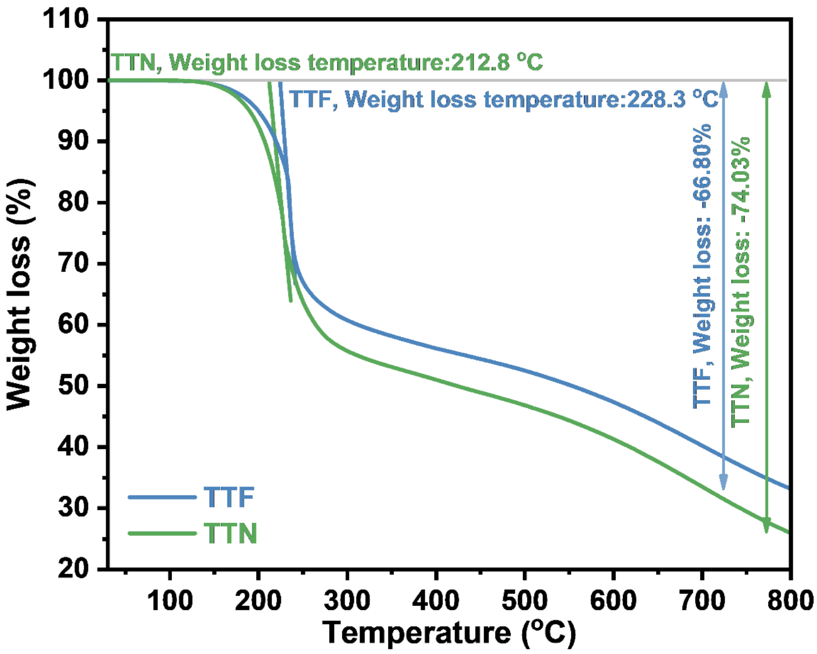


**Fig. S11** TGA and DTG curves of **a** TTF and **b** TTN. Compared with TTN, TTF had higher thermal stability. The weight loss starting temperature of TTF was 228.3 °C, which was higher than the weight loss starting temperature of TTN 212.8 °C, and when the temperature raised to 800 °C, the weight loss ratio of TTF was 66.80%, while the weight loss ratio of TTN was 74.03%. The higher stability of TTF may be ascribed to the more planar structure

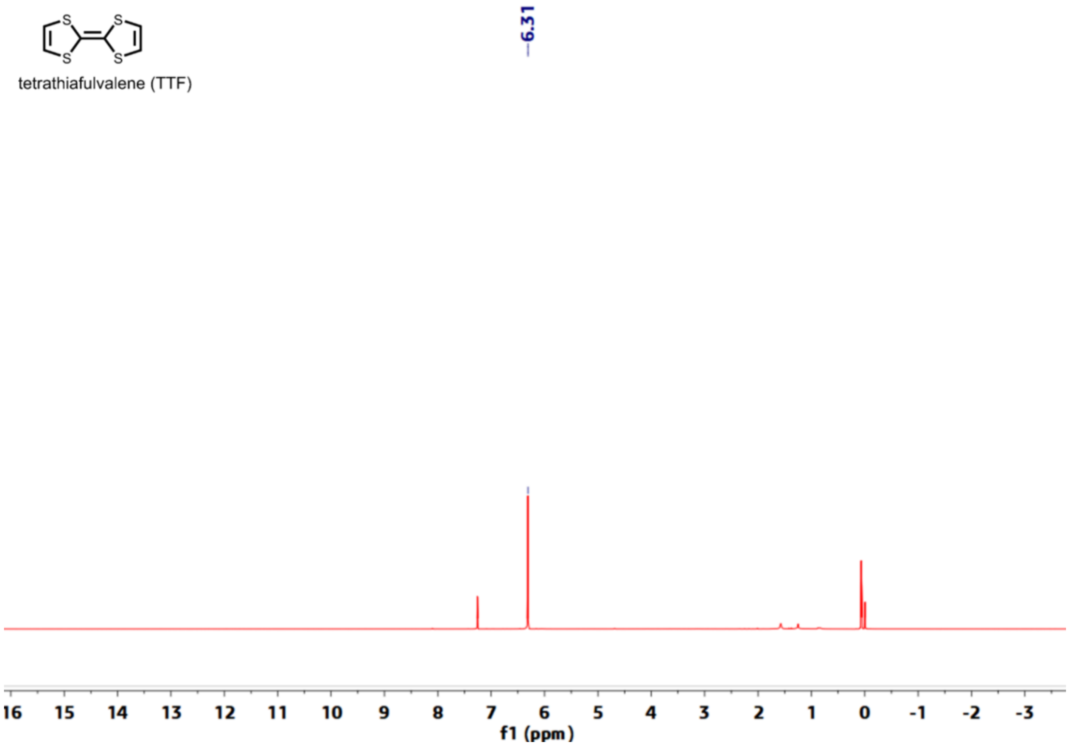


**Fig. S12** $^1$H NMR (600 MHz, $CDCl_3$, 300 K) spectra of TTF

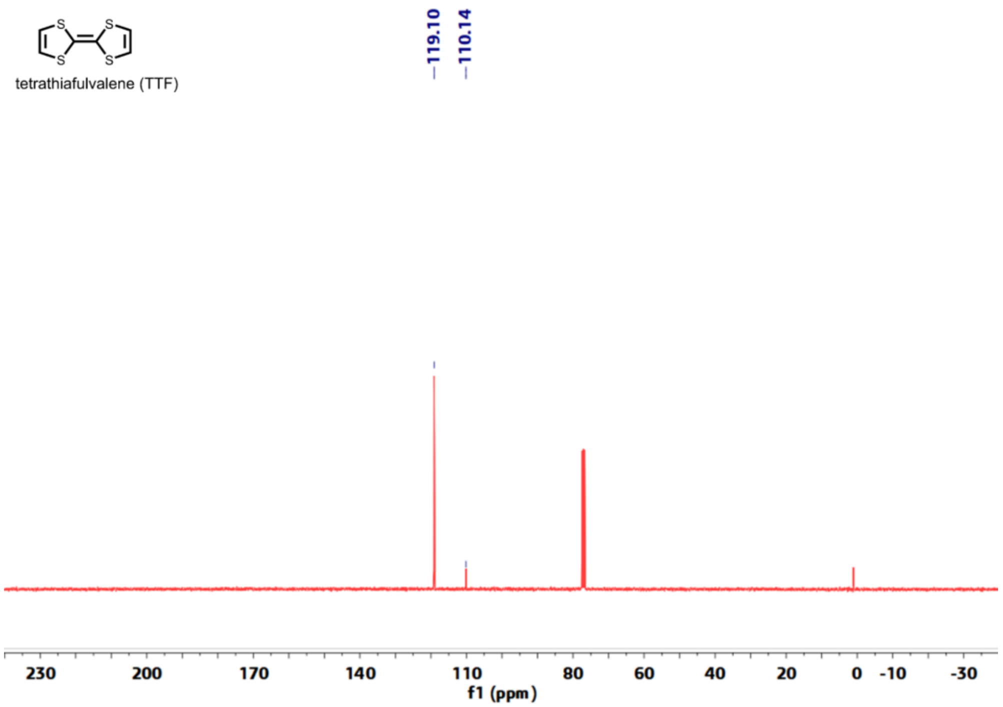


**Fig. S13** $^{13}$C NMR (600 MHz, $CDCl_3$, 300 K) spectra of TTF

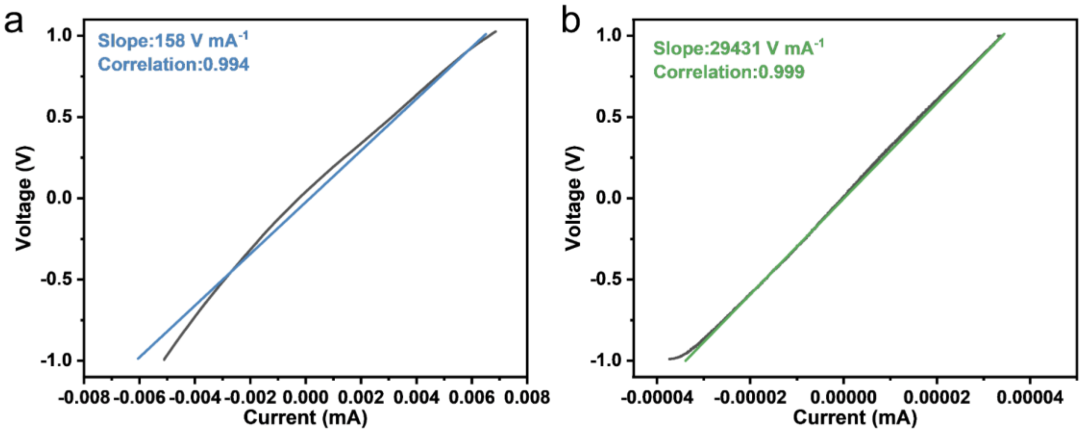


**Fig. S14** The I-V curves of **a** TTF and **b** TTN

**Table S1** The electrical conductivity for two isomers

| Sample | R (Ω) | d (mm) | S ($cm^2$) | σ (S/cm) |
|---|---|---|---|---|
| TTF | $1.58\times10^5$ | 0.21 | 1.131 | $1.18\times10^{-6}$ |
| TTN | 2.94×107 | 0.34 | 1.131 | $1.02\times10^{-8}$ |

The electrical conductivity was measured using linear sweep voltammetry via BioLogic VMP3 potentiostat. The powder was pressed into pellet at the pressure of 20 MPa, and the pellets were sandwiched between two stainless steel gaskets for test. The electrical conductivity was calculated by the formula:

$$\sigma = \frac{d}{R \times S}$$

where R equalled the slope of the I-V curve, and S and d were the area and thickness of the pellet, respectively.

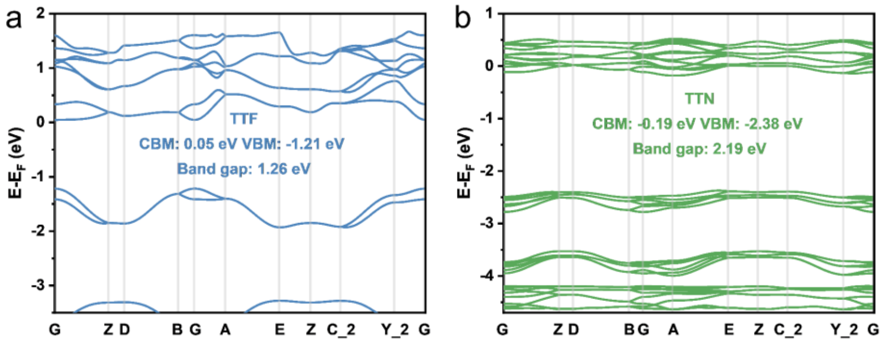


**Fig. S15** Electronic band structure of **a** TTF and **b** TTN by using the crystal structures. The calculated (conduction band minimum) CBM energy and (Valence Band Maximum) VBM energy of TTF and TTN were 0.05 eV, -1.21 eV, -0.19 eV and -2.38 eV, respectively, and the band gap of TTF (1.26 eV) was narrower than that of TTN (2.19 eV). The trends of these results were the same as those exhibited by the calculated HOMO/LUMO energy levels

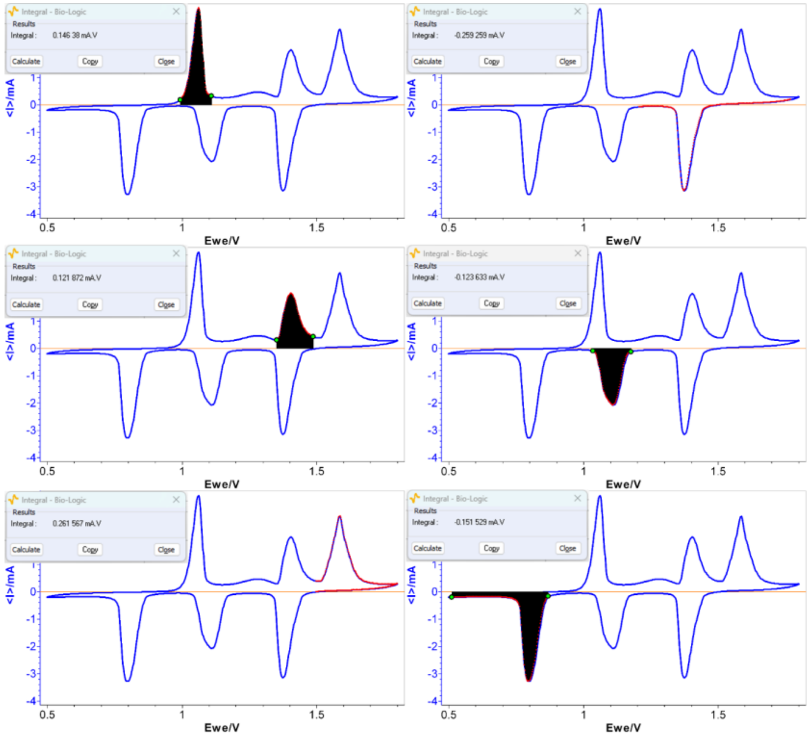


**Fig. S16** The integral areas for the redox peaks of TTF electrode. TTF electrode showed three pairs of redox peaks with ratio of the integral areas for the three pairs of peaks around 1:1:2, indicating that two electrons in total were involved for every TTF molecule during the cycling and the first two redox peaks involved one electron transfer and the third redox peak involved another electron transfer

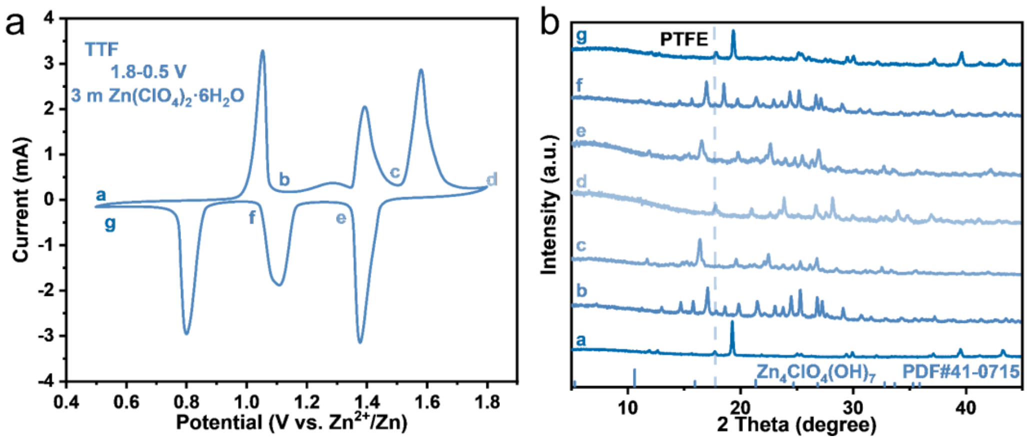


**Fig. S17 a** XRD tests were conducted at the marked points. **b** XRD patterns of TTF electrodes under different voltages. The results indicated that TTF underwent a reversible phase transition during the ion intercalation/extraction process

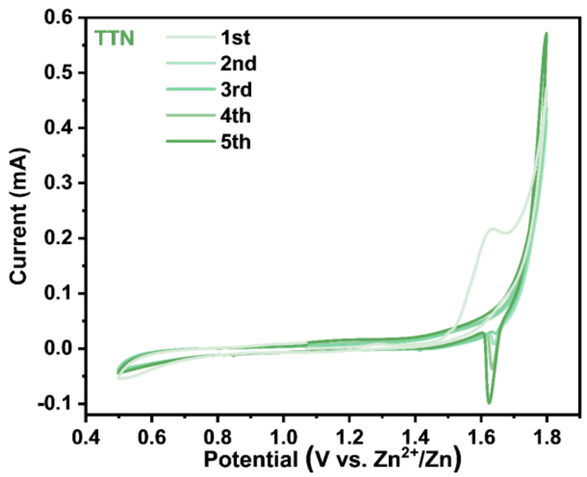


**Fig. S18** CV curves of TTN electrode at 1 mV $s^{-1}$ in 3 m $Zn(ClO_4)_2 \cdot 6H_2O$ electrolyte and the voltage range of 0.5-1.8 V

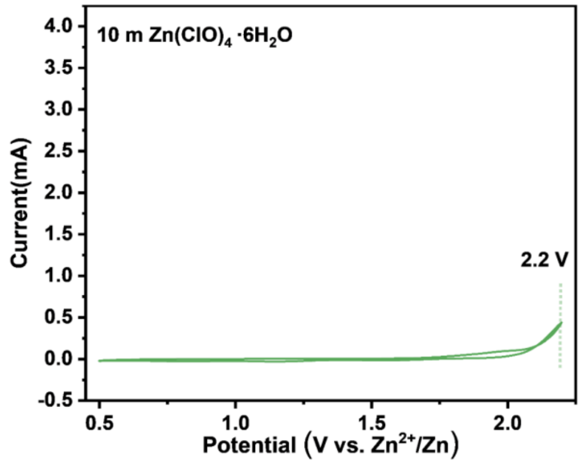


**Fig. S19** CV curves of stainless steel meshes//Zn cell at 1 mV $s^{-1}$ in 10 m $Zn(ClO_4)_2 \cdot 6H_2O$ electrolyte and the voltage range of 0.5-2.2 V, showing slight degradation of the electrolyte higher than 2.1 V

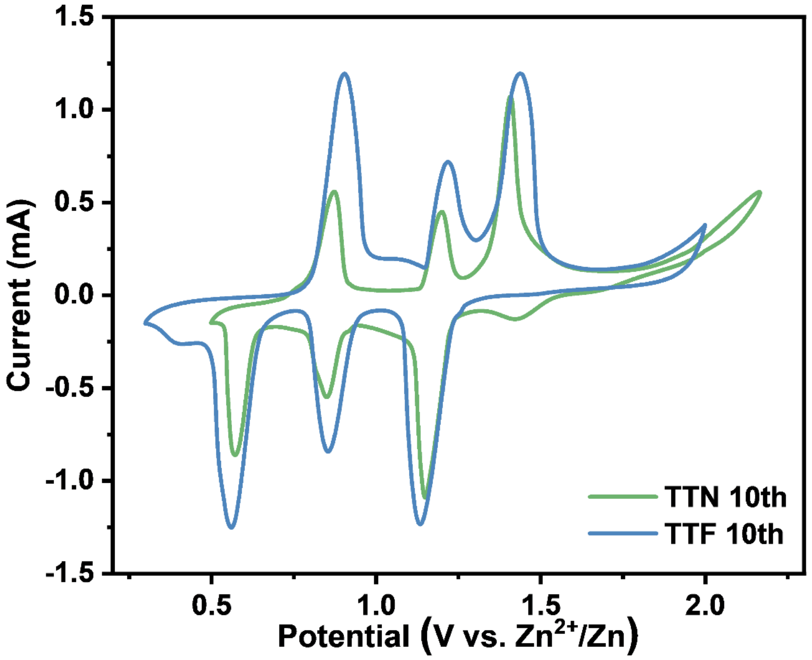


**Fig. S20** Comparison of CV Curves of TTN and TTF electrodes after stabilization at 1 mV $s^{-1}$ in 10 m $Zn(ClO_4)_2 \cdot 6H_2O$ electrolyte, showing TTN was converted into TTF

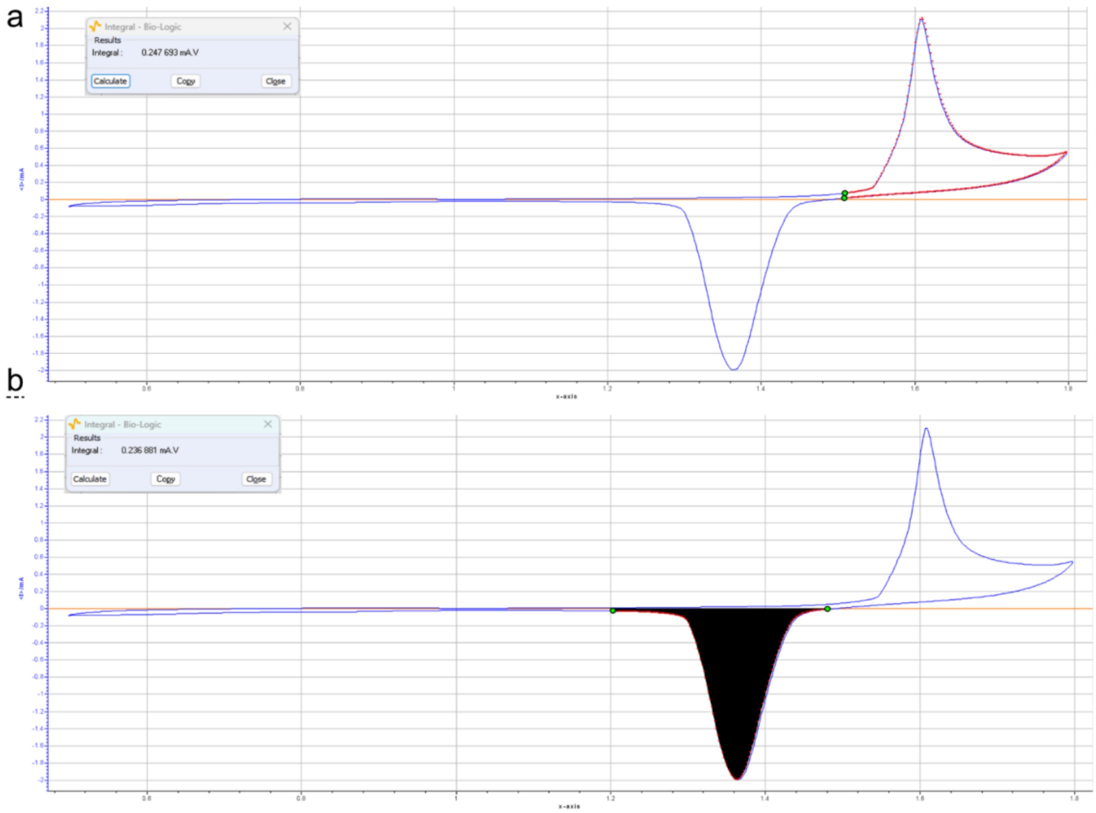


**Fig. S21** The integral areas for the redox peaks of TTN electrode in 10 m $Zn(ClO_4)_2 \cdot 6H_2O$ electrolyte and the voltage range of 0.5-1.8 V

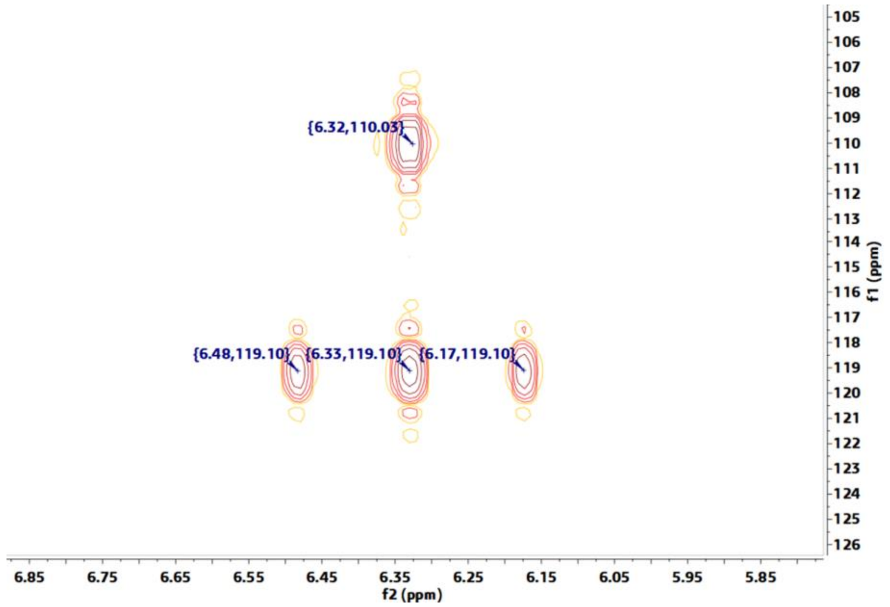


**Fig. S22** HMBC spectra of TTF

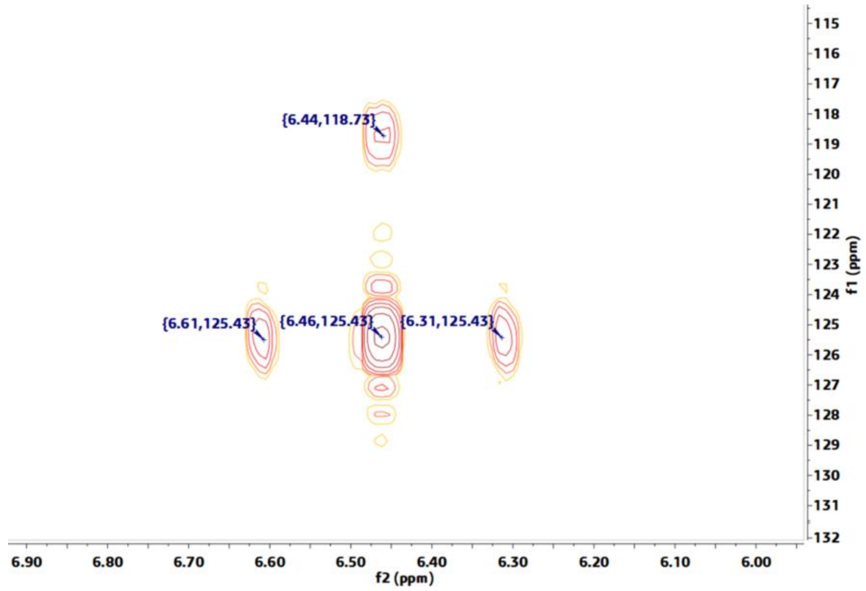


**Fig. S23** HMBC spectra of TTN

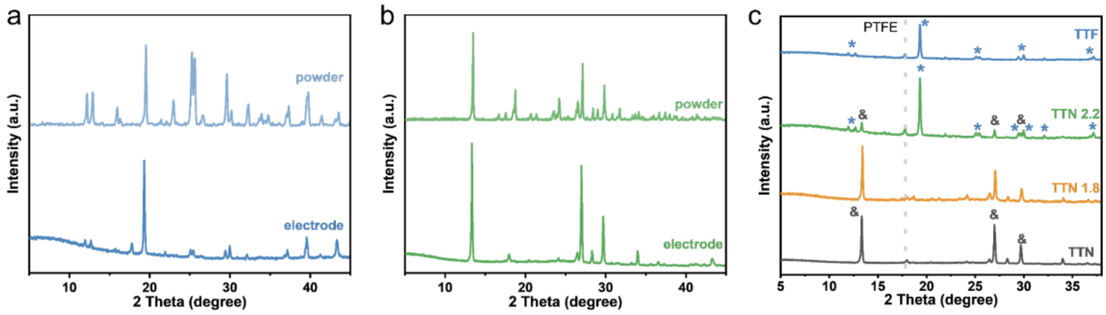


**Fig. S24** Comparison of XRD patterns of TTF and TTN power with their electrode. **a** TTF. **b** TTN. **c** Ex-situ XRD patterns of TTN, TTN 1.8, TTN 2.2 and TTF electrodes

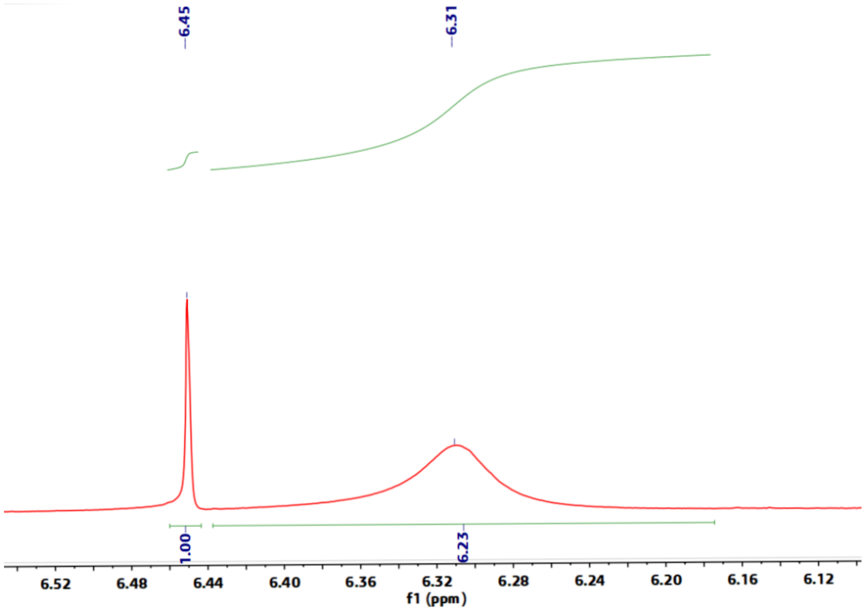


**Fig. S25** Ex-situ $^{1}$H NMR spectrum of TTN 2.2. The conversion rate calculated through integral proportion was about 86%

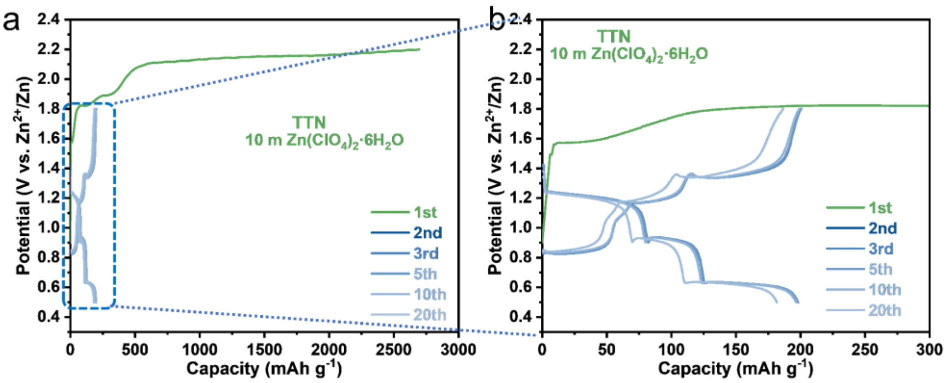


**Fig. S26 a** Charge-discharge profile of TTN electrode at 131 mA g$^{-1}$ in 10 m $Zn(ClO_4)_2 \cdot 6H_2O$ electrolyte. The cut-off voltage was set at 2.2 V for the first charge, and the subsequent cut-off voltage was set at 1.8 V. **b** Partial enlarged view of charge-discharge profile. We performed galvanostatic charge/discharge tests of TTN electrodes, with the cut-off voltage set at 2.2 V for the first charge and the subsequent cut-off voltage at 1.8 V. As shown in Fig. S26, the first charge showed abnormally high capacity, which should be ascribed to the decomposition of the electrolyte. However, under such high voltage, the TTN was converted into TTF. Afterwards, the galvanostatic charge/discharge tests of TTN electrodes showed similar curves with those of TTF electrodes in the shapes, three plateaus as well as the reversible discharge capacity of 200 mAh g$^{-1}$ (capacity utilization of 76%, based on two-electron transfer)

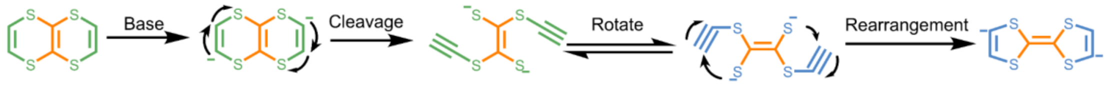


**Scheme S2** The process of converting TTN to TTF through deprotonation under the action of base

**Discussion S1: Calculation of potential downshift based on Nernst Equation**

The electrochemical reaction of p-type TTN electrode in aqueous $Zn(ClO_4)_2·6H_2O$ could be expressed as below:

$$TTN+ClO_4^- \leftrightarrow TTN^+ClO_4^- + e^- \quad (S1)$$

Nernst Equation:

$$E_{TTN}^{TTN^+} = E^{0,}{}_{TTN}^{TTN^+} + 2.303\frac{RT}{F}\times\log\left(\frac{1}{\alpha_{ClO_4^-}}\right) \quad (S2)$$

Where E was electrode potential; $E^0$ was the standard potential; R was the ideal gas constant: 8.314 J $K^{-1}$ $mol^{-1}$; T was the temperature: 298.15 K; F was the Faraday constant: 96500 C $mol^{-1}$; $\alpha_{ClO_4^-}$ was the activity of the anion.

Therefore, the redox potential of TTN in the high-concentration $Zn(ClO_4)_2·6H_2O$ aqueous electrolyte should shift downward more than that in the low-concentration one, as indicated by equation (2), owing to the increased activity of the anion ($\alpha_{ClO_4^-}$), which was proportional to the mole concentration. In addition, a higher concentration of electrolyte could also inhibit the evolution of oxygen, which was beneficial to expand the electrochemical window of the electrolyte.

**Discussion S2: Replacing the active hydrogen on TTN still could not prevent the occurrence of molecular rearrangement**

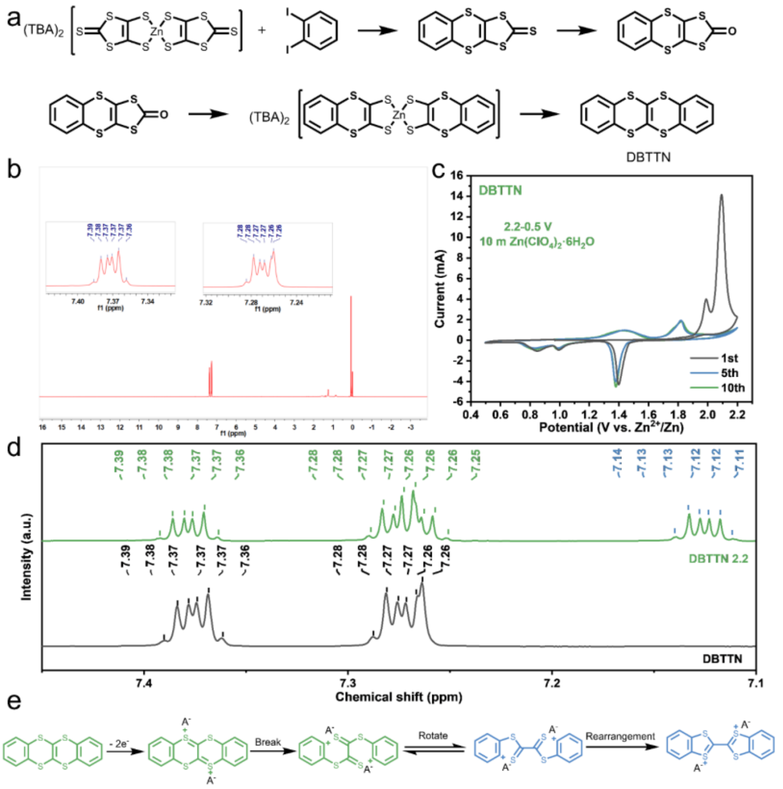


**Fig. S27 a** Synthetic process of DBTTN. **b** $^1H$ NMR (600 MHz, $CDCl_3$, 300 K) spectra of DBTTN. **c** CV curves of DBTTN electrode at 1 mV $s^{-1}$ in 10 m $Zn(ClO_4)_2·6H_2O$

electrolyte and the voltage range of 0.5-2.2 V. **d** Ex-situ $^1$H NMR spectra of DBTTN 2.2. **e** The proposed process of solid-state molecular rearrangement of DBTTN to DBTTF at high voltage

If the conversion process from TTN to TTF was the same as that of base induce deprotonation rearrangement, the designed fused-TTN (DBTTN) should not experience isomerization at high voltage (or further oxidation), due to the absence of hydrogen atoms at the ethene bridges. The synthesis process of DBTTN was shown in Fig. 27a, and the $^1$H NMR spectra of DBTTN was consistent with the literature [S2], proving the accurate synthesis of the target product (Fig. S27b). We first assembled DBTTN//Zn cell with 10 m $Zn(ClO_4)_2 \cdot 6H_2O$ as electrolyte, and carried out cyclic voltammetry test on it in the voltage range of 0.5-2.2 V. As shown in Fig. S27c, the DBTTN electrode exhibited two strong oxidation peaks at 1.99 V and 2.09 V when first charged. However, during subsequent charging, two new peaks at 1.44 V and 1.82 V, respectively, appeared, in place of the two peaks observed during the first charging. In addition, in each discharge process, there were three reduction peaks at 1.39 V, 0.99 V and 0.85 V, which had a large polarization relative to the oxidation peak of the first charged. This redox behavior was very similar to that of TTN electrodes, so we performed $^1$H NMR test on DBTTN after the first charge and then discharge (defined as DBTTN 2.2). As shown in Fig. S27d, compared with the pristine DBTTN, the DBTTN 2.2 exhibited three sets of different chemical shift signals. Among them, the chemical shifts in the range of 7.39-7.36 ppm were consistent with the pristine DBTTN, indicating that the pristine state of DBTTN still existed. However, some additional peaks appeared in the range of 7.14–7.11 ppm and 7.28-7.26 ppm, which belonged to fused-TTF (DBTTF) [S3].This result indicated that the isomerization process from TTN to TTF under high voltage (or further oxidation) was different from the process of base induced deprotonation rearrangement. The proposed isomerization process may be more reasonable (Fig. S27e).

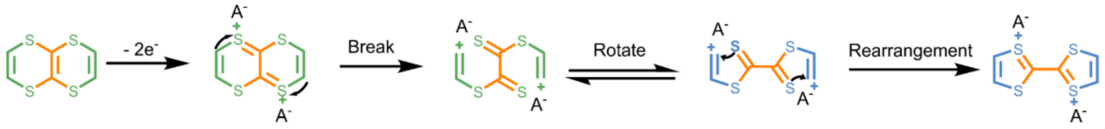


**Scheme S3** The proposed process of solid-state molecular rearrangement of TTN to TTF

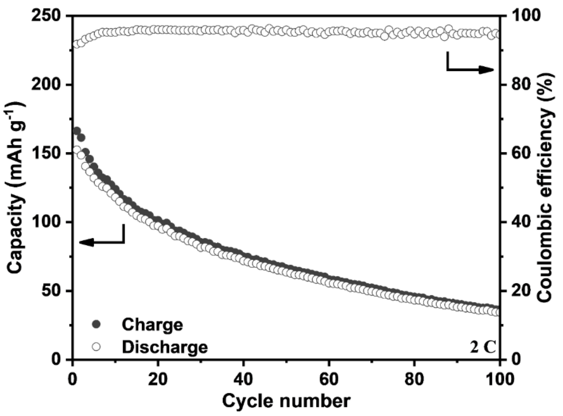


**Fig. S28** Cycling stability of TTF electrode at a current density of 2 C when Super P was used as conductive additive. TTF electrode exhibited low initial specific capacity and poor cycling stability

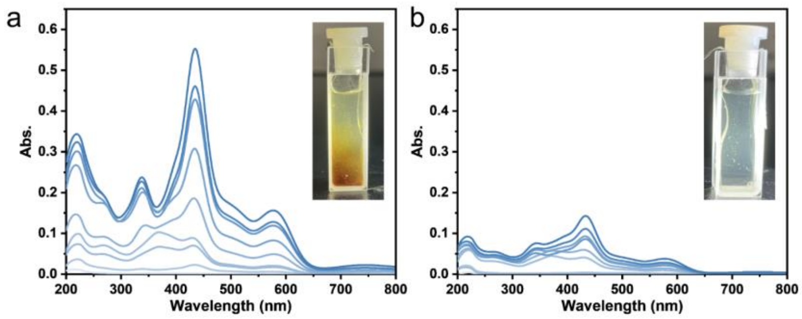


**Fig. S29** In-situ UV-vis spectra of TTF electrode for **a** Super P as conductive additive and **b** rGO as conductive additive. The insets were the images of the cuvette cell after 10 cycles. It could be clearly observed that when Super P was used as conductive additive, several peaks belonging to TTF derivatives increases significantly with cycling and the color of electrolyte solution changed to orange, indicating that TTF derivatives were dissolved in the electrolyte. However, when rGO was used as conductive additive, the adsorption peak of TTF derivatives were extremely weak and the color of electrolyte showed insignificant change

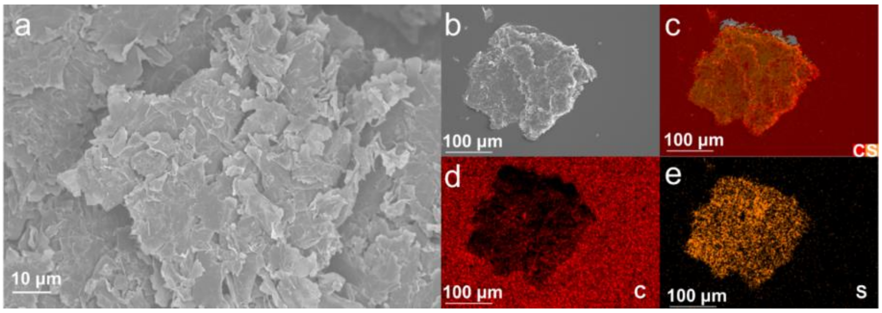


**Fig. S30** **a** SEM image of the mixture of TTF and rGO. **b-e** Corresponding elemental C and S mapping. The uniform mixing of TTF and rGO could be proved by SEM images and corresponding element mapping, which was conducive to the transport of ions and electrons and could effectively inhibit the dissolution of intermediates

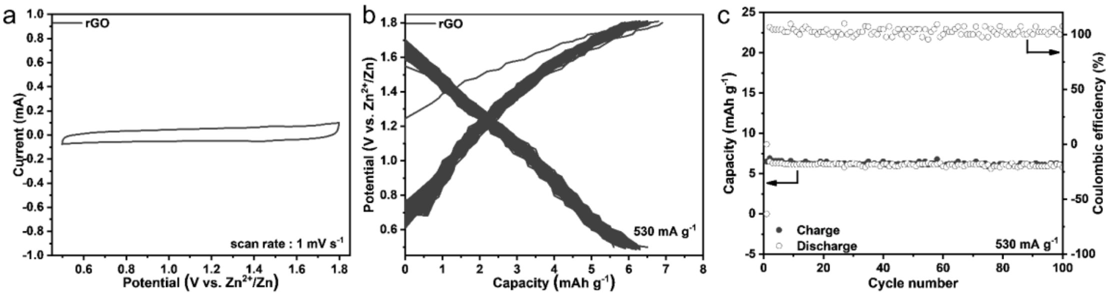


**Fig. S31 a** CV curves of rGO electrode at 1 mV s$^{-1}$ in 3 m $Zn(ClO_4)_2 \cdot 6H_2O$ electrolyte. **b** charge-discharge profile of rGO electrode at 0.53 A g$^{-1}$. **c** the cycling performance of rGO electrode at 0.53 A g$^{-1}$

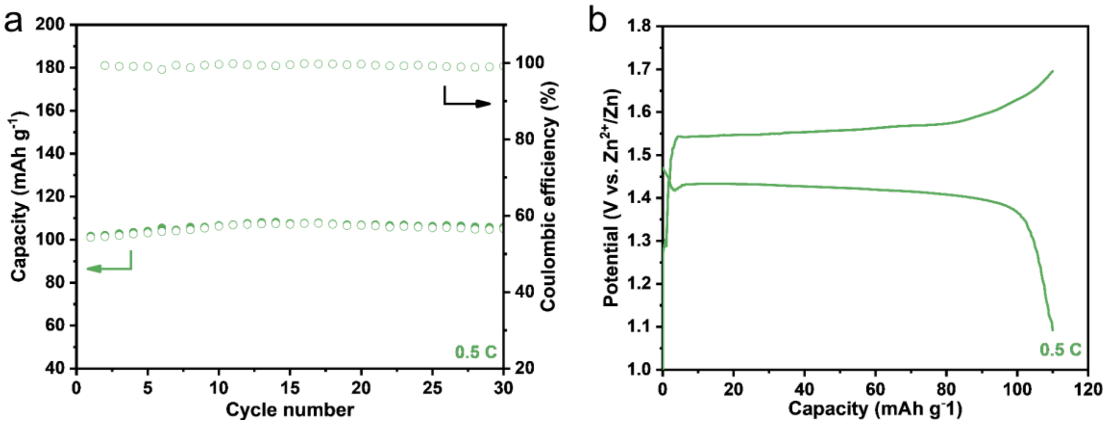


**Fig. S32 a** Cycling performance of TTN electrode at 0.5 C. **b** Charge-discharge profile of TTN electrode at 0.5 C

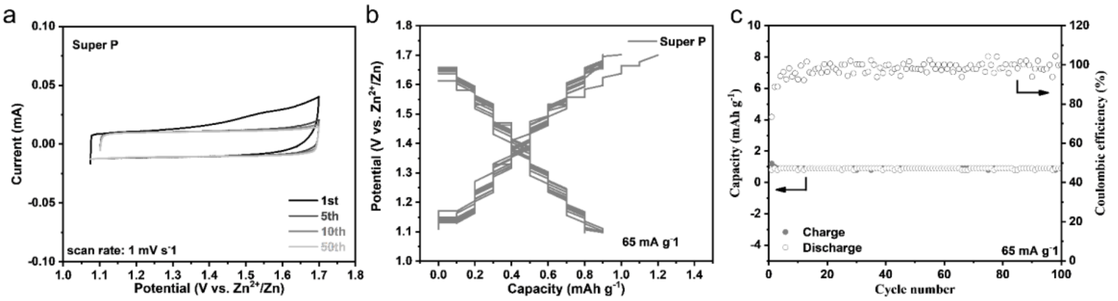


**Fig. S33 a** CV curves of Super P electrode at 1 mV s$^{-1}$ in 10 m $Zn(ClO_4)_2 \cdot 6H_2O$ electrolyte. **b** charge-discharge profile of Super P electrode at 0.065 A g$^{-1}$. **c** the cycling performance of Super P electrode at 0.065 A g$^{-1}$. Although there were some reports that carbon materials such as graphite had the ability to store anions, high voltage (>2 V) was required [S4, S5]. In this work, Super P had almost no capacity contribution

**Table S2** Electrochemical performance comparison of reported organic electrodes for aqueous rechargeable zinc batteries

| Materials | Storage of possible ion types | Capacity, Current density (mAh $g^{-1}$, A $g^{-1}$) | Average discharge voltages (V) | Energy density (Wh $kg^{-1}$) | Refs. |
|---|---|---|---|---|---|
| 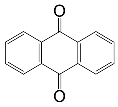 9,10-AQ | $Zn^{2+}$ | ~180, 0.02 | ~0.5 | - | [S6] |
| 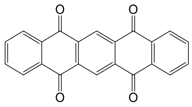 PT | $Zn^{2+}$ | ~170, 0.08 | ~0.6 | ~102 | [S7] |
| 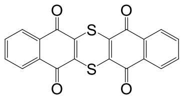 DTT | $Zn^{2+}$, $H^+$ | 200, 0.1 | ~0.63 | ~126.5 | [S8] |
| 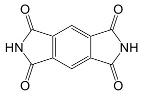 NDI | $Zn^{2+}$, $H^+$ | ~120, 0.02 | ~0.55 | ~66 | [S9] |
| 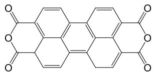 PTCDA | $Zn^{2+}$ | 122.9, 0.2 | ~0.52 | ~62 | [S10] |
| 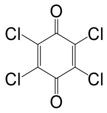 p-Chloranil/CMK-3 | $Zn^{2+}$ | ~170, 0.043 | ~1.1 | ~187 | [S11] |
| 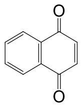 NQ/CNT | $Zn^{2+}$ | 250, 0.339 | ~0.6 | - | |
| 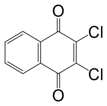 2Cl-NQ/CNT | $Zn^{2+}$ | ~210, 0.118 | ~0.75 | - | [S12] |
| 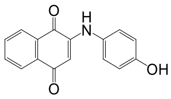 | $Zn^{2+}$ | ~200, 0.151 | ~0.58 | - | |

| | | | | | |
|---|---|---|---|---|---|
| APh-NQ/CNT | | | | | |
| 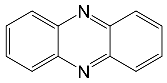 PNZ | $Zn^{2+}$ | 265, 0.02 | ~0.9 | ~238 | [S13] |
| 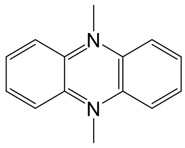 DMPZ | $ClO_4^-$ | 231, 0.51 | 1.0 | ~231 | [S14] |
| 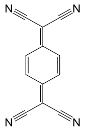 TCNQ | $Zn^{2+}$ | 123.2, 0.1 | ~0.95 | ~117 | [S15] |
| 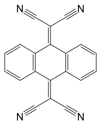 TCNAQ | $Zn^{2+}$ | 169, 0.02 | ~0.9 | ~151 | [S16] |
| 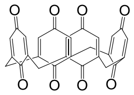 C4Q | $Zn^{2+}$ | 150, 0.5 | ~1.07 | ~335 | [S6] |
| 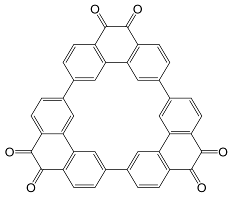 PQ | $Zn^{2+}$ | 203, 0.03 | ~0.89 | ~180 | [S17] |
| 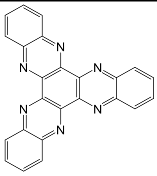 HATN | $H^+$ | 175, 1.0 | ~0.52 | ~192.4 | [S18] |
| 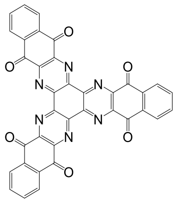 HATNQ | $Zn^{2+}$, $H^+$ | 245, 5.0 | ~0.6 | ~289 | [S19] |

| | | | | | |
|---|---|---|---|---|---|
| 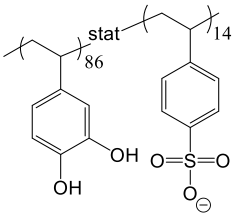 (P(4VC$_{86}$-stat-SS$_{14}$)) | $Zn^{2+}$ | 274, 3.4 | 1.1 | ~301 | [S20] |
| 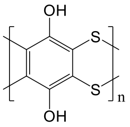 PTFHQ | $Zn^{2+}$, $H^{+}$ | 215, 0.1 | 1.0 | ~215 | [S21] |
| 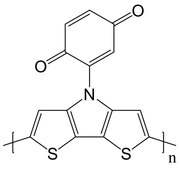 PDpBQH | $Zn^{2+}$, $H^{+}$ | 120, 0.1 | ~1.1 | ~121 | [S22] |
| 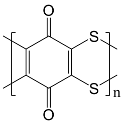 PDB | $Zn^{2+}$ | ~100, 0.047 | ~0.85 | ~85 | [S23] |
| 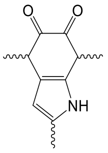 PDA/CNT | $Zn^{2+}$ | 126, 0.02 | ~0.8 | ~100.9 | [S24] |
| 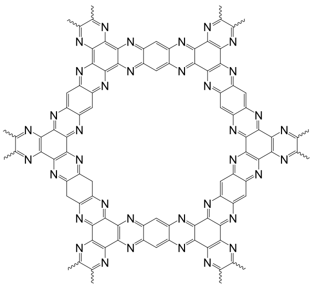 HA-COF | $Zn^{2+}$, $H^{+}$ | 164, 0.1 | ~0.7 | ~115 | [S25] |
| 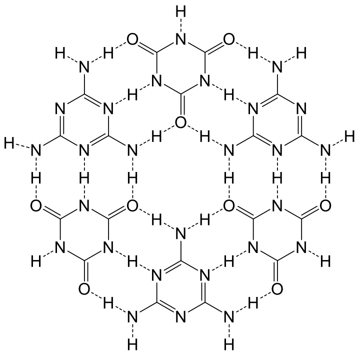 HBOS | $H^{+}$ | 298, 2.0 | 0.86 | ~256 | [S26] |

| | | | | | |
|---|---|---|---|---|---|
| 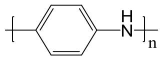 PANI | $Zn^{2+}$, $CF_3SO_3^-$ | ~200, 0.05 | ~0.86 | ~171 | [S27] |
| 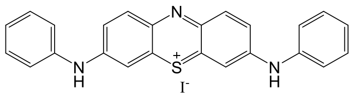 PTD-1 | $H^+$, $SO_4^{2-}$ | ~130, 0.2 | ~1 | ~116.8 | [S28] |
| 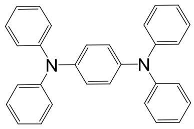 BDB | $TFSI^-$ /$CF_3SO_3^-$ | ~112, 0.39 | ~1.1 | ~123 | [S29] |
| 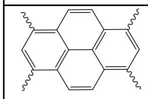 CLPy | $Cl^-$ | 180, 0.05 | ~0.84 | ~151 | [S30] |
| 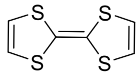 TTF | $ClO_4^-$ | 220, 0.52 | 1.05 | ~213 | This work |
| 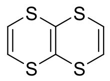 TTN | $ClO_4^-$ | 85, 0.26 | 1.42 | ~120 | This work |

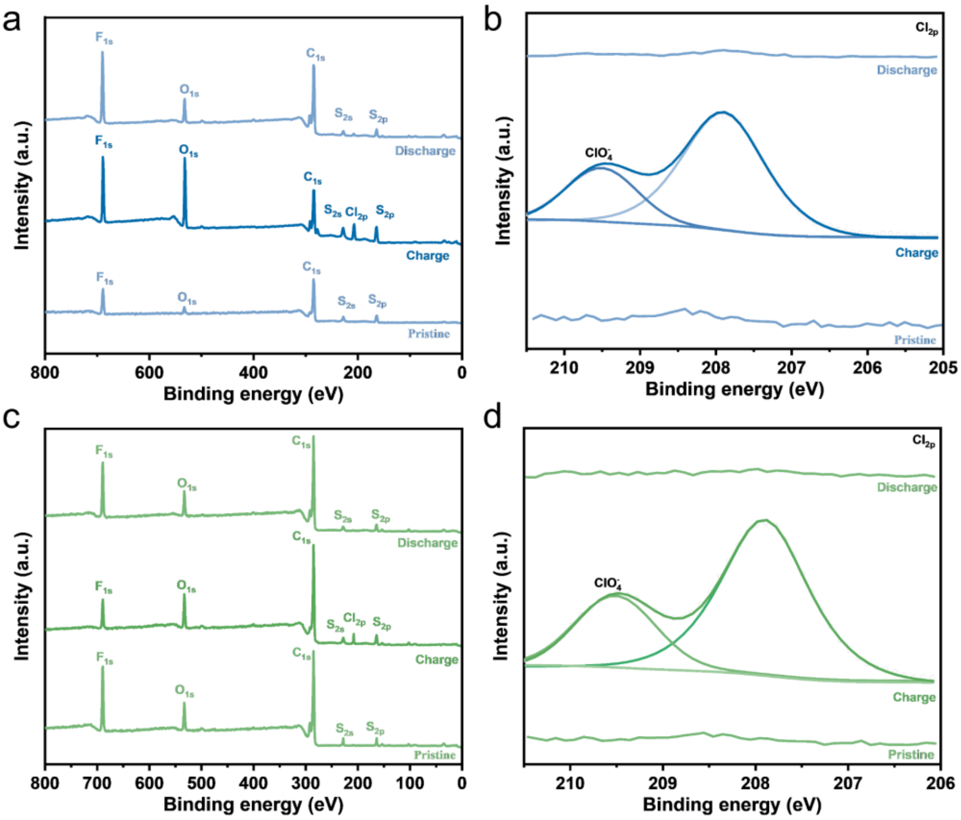


**Fig. S34 a** XPS spectra of TTF electrode in pristine, completely charged and completely discharged state, respectively. **b** High-resolution Cl 2p XPS spectra of TTF electrode in

pristine, completely charged and completely discharged state, respectively. **c** XPS spectra of TTN electrode in pristine, completely charged and completely discharged state, respectively. **d** High-resolution Cl 2p XPS spectra of TTN electrode in pristine, completely charged and completely discharged state, respectively

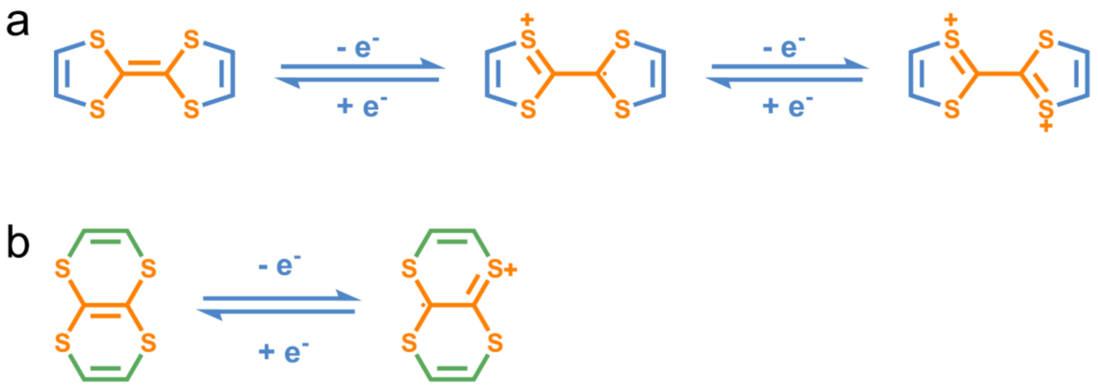


**Scheme S4 a** Possible redox mechanism of TTF. When TTF was oxidized, the resonance structures involved in the formation of the radical cation, and further oxidation to form the dication species. **b** Possible redox mechanism of TTN. TTN could only be stably oxidized into monovalent free radical cations

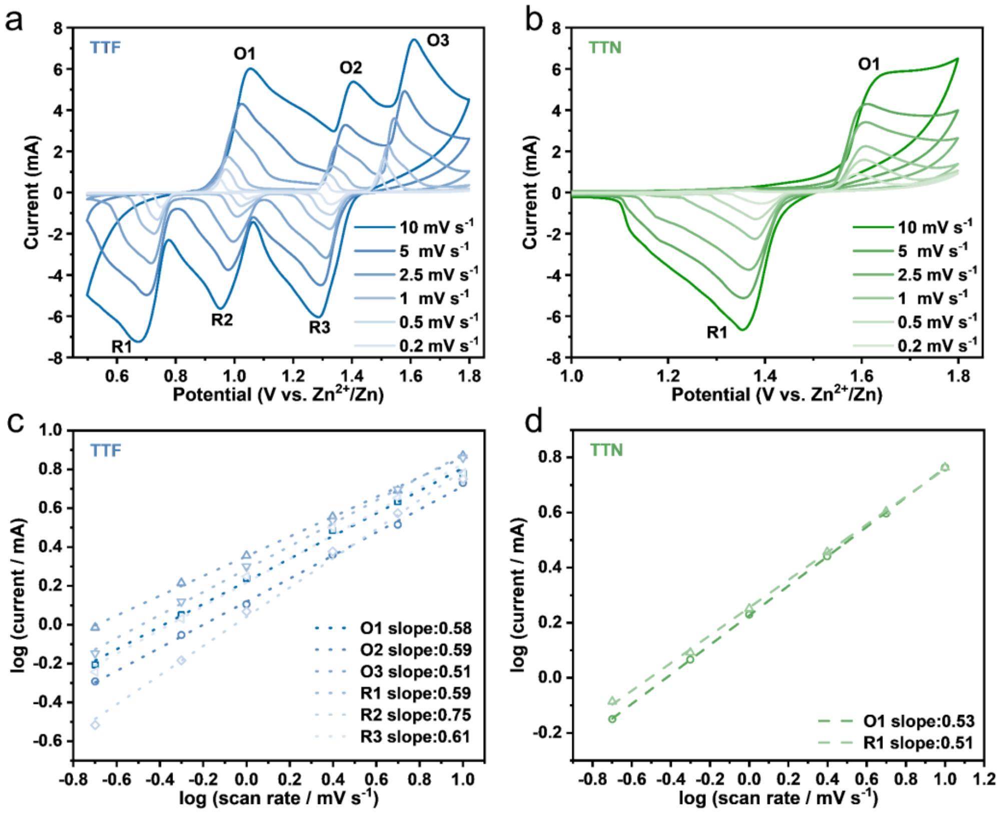


**Fig. S35 a-b** CV curves of TTF and TTN electrode at different scan rates from 0.2 to 10 mV $s^{-1}$. **c-d** Plots of log (i, mA) versus log (v, mV $s^{-1}$) by using the corresponding peak currents in the CV curves of TTF and TTN electrode at the different scan rates

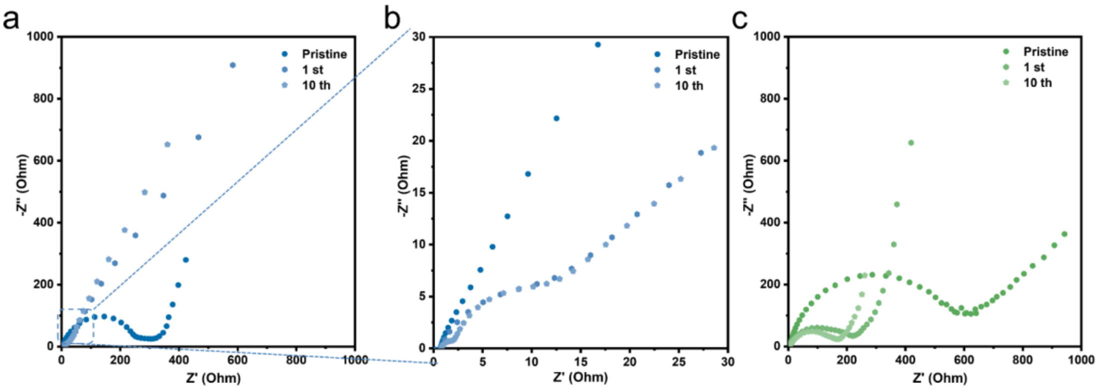


**Fig. S36 a** Nyquist plots of TTF electrode and **b** Partial enlargement after different numbers of cycles. **c** Nyquist plots of TTN electrode after different numbers of cycles. The resistance of TTF and TTN electrode after discharged ten cycles was ~20 Ω and ~170 Ω, respectively. The TTF electrode showed lower resistance, further confirming its faster charge transfer for achieving high-rate performance

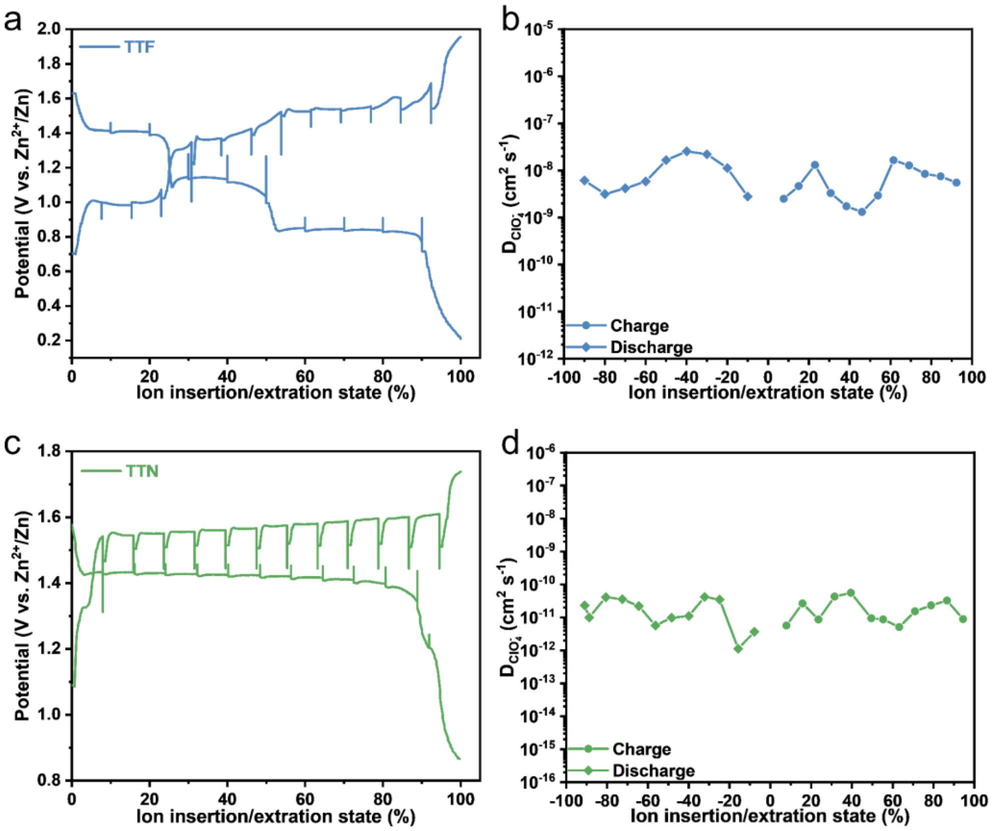


**Fig. S37 a** The GITT curves of TTF electrode. **b** The ion diffusion coefficient of the TTF electrode. **c** The GITT curves of TTN electrode. **d** The ion diffusion coefficient of the TTN electrode

## Supplementary References

[S1] T.K. Hansen, J. Becher, T. Jørgensen, K.S. Varma, R. Khedekar et al., 4,5-dibenzoyl-1,3-dithiole-1-thione. Organic Synth. **73**, 270 (1996).

https://doi.org/10.15227/orgsyn.073.0270

[S2] Y. Sun, Z. Cui, L. Chen, X. Lu, Y. Wu et al., Aryl-fused tetrathianaphthalene (ttn): Synthesis, structures, properties, and cocrystals with fullerenes. RSC Adv. **6**, 79978 (2016). https://doi.org/10.1039/c6ra18945h

[S3] N. Sakai, K. Minato, S. Nakata, Y. Ogiwara, Synthesis of dibenzotetrathiafulvalenes of oxalic acid with electron-rich aromatic 1,2-dithiols and application to dithioacetalization with 9-fluorenecarboxylic acids or dicarboxylic acids. Synthesis **54**, 2661 (2022). https://doi.org/10.1055/a-1742-2821

[S4] Z. Huang, Y. Hou, T. Wang, Y. Zhao, G. Liang et al., Manipulating anion intercalation enables a high-voltage aqueous dual ion battery. Nat. Commun. **12**, 3106 (2021). https://doi.org/10.1038/s41467-021-23369-5

[S5] H. Zhang, X. Liu, B. Qin, S. Passerini, Electrochemical intercalation of anions in graphite for high-voltage aqueous zinc battery. J. Power Sources **449**, 227594 (2020). https://doi.org/10.1016/j.jpowsour.2019.227594

[S6] Q. Zhao, W.W. Huang, Z.Q. Luo, L.J. Liu, Y. Lu et al., High-capacity aqueous zinc batteries using sustainable quinone electrodes. Sci. Adv. **4**, eaao1761 (2018). https://doi.org/10.1126/sciadv.aao1761

[S7] C. Mirle, V. Medabalmi, K. Ramanujam, Electrode and conductive additive compatibility yielding excellent rate capability and long cycle life for sustainable organic aqueous zn-ion batteries. ACS Appl. Energy Mater. **4**, 1218 (2021). https://doi.org/10.1021/acsaem.0c02511

[S8] Y. Wang, C. Wang, Z. Ni, Y. Gu, B. Wang et al., Binding zinc ions by carboxyl groups from adjacent molecules toward long-life aqueous zinc-organic batteries. Adv. Mater. **32**, e2000338 (2020). https://doi.org/10.1002/adma.202000338

[S9] M. Na, Y. Oh, H.R. Byon, Effects of zn2+ and h+ association with naphthalene diimide electrodes for aqueous zn-ion batteries. Chem. Mater. **32**, 6990 (2020). https://doi.org/10.1021/acs.chemmater.0c02357

[S10] H. Zhang, Y. Fang, F. Yang, X. Liu, X. Lu, Aromatic organic molecular crystal with enhanced π–π stacking interaction for ultrafast zn-ion storage. Energy Environ. Sci. **13**, 2515 (2020). https://doi.org/10.1039/d0ee01723j

[S11] D. Kundu, P. Oberholzer, C. Glaros, A. Bouzid, E. Tervoort et al., Organic cathode for aqueous zn-ion batteries: Taming a unique phase evolution toward stable electrochemical cycling. Chem. Mater. **30**, 3874 (2018). https://doi.org/10.1021/acs.chemmater.8b01317

[S12] J. Kumankuma-Sarpong, S. Tang, W. Guo, Y. Fu, Naphthoquinone-based composite cathodes for aqueous rechargeable zinc-ion batteries. ACS Appl.

Mater. Interfaces **13**, 4084 (2021). https://doi.org/10.1021/acsami.0c21339

[S13] S. Zhang, S. Long, H. Li, Q. Xu, A high-capacity organic cathode based on active n atoms for aqueous zinc-ion batteries. Chem. Eng. J. **400**, 125898 (2020). https://doi.org/10.1016/j.cej.2020.125898

[S14] M.H. Lee, G. Kwon, H. Lim, J. Kim, S.J. Kim et al., High-energy and long-lasting organic electrode for a rechargeable aqueous battery. ACS Energy Lett. **7**, 3637 (2022). https://doi.org/10.1021/acsenergylett.2c01535

[S15] N.M. Chola, R.K. Nagarale, Tcnq confined in porous organic structure as cathode for aqueous zinc battery. J. Electrochem. Soc. **167**, 100552 (2020). https://doi.org/10.1149/1945-7111/ab9cc9

[S16] Q. Wang, X. Xu, G. Yang, Y. Liu, X. Yao, An organic cathode with tailored working potential for aqueous zn-ion batteries. Chem. Commun. **56**, 11859 (2020). https://doi.org/10.1039/d0cc05344a

[S17] K.W. Nam, H. Kim, Y. Beldjoudi, T.W. Kwon, D.J. Kim et al., Redox-active phenanthrenequinone triangles in aqueous rechargeable zinc batteries. J. Am. Chem. Soc. **142**, 2541 (2020). https://doi.org/10.1021/jacs.9b12436

[S18] Z. Tie, L. Liu, S. Deng, D. Zhao, Z. Niu, Proton insertion chemistry of a zinc-organic battery. Angew. Chem. Int. Ed. **59**, 4920 (2020). https://doi.org/10.1002/anie.201916529

[S19] Y. Chen, J. Li, Q. Zhu, K. Fan, Y. Cao et al., Two-dimensional organic supramolecule via hydrogen bonding and π–π stacking for ultrahigh capacity and long-life aqueous zinc-organic batteries. Angew. Chem. Int. Ed. **61**, 202116289 (2022). https://doi.org/10.1002/anie.202116289

[S20] N. Patil, C. Cruz, D. Ciurduc, A. Mavrandonakis, J. Palma et al., An ultrahigh performance zinc-organic battery using poly(catechol) cathode in zn(tfsi)2-based concentrated aqueous electrolytes. Adv. Energy Mater. **11**, 2100939 (2021). https://doi.org/10.1002/aenm.202100939

[S21] Q. Sun, T. Sun, J.Y. Du, Z.L. Xie, D.Y. Yang et al., In situ electrochemical activation of hydroxyl polymer cathode for high‐performance aqueous zinc–organic batteries. Angew. Chem. Int. Ed. e202307365 (2023). https://doi.org/10.1002/anie.202307365

[S22] X. Wang, J. Xiao, W. Tang, Hydroquinone versus pyrocatechol pendants twisted conjugated polymer cathodes for high‐performance and robust aqueous zinc‐ion batteries. Adv. Funct. Mater. **32**, 2108225 (2021). https://doi.org/10.1002/adfm.202108225

[S23] W. Yang, X. Du, J. Zhao, Z. Chen, J. Li et al., Hydrated eutectic electrolytes with ligand-oriented solvation shells for long-cycling zinc-organic batteries.

Joule **4**, 1557 (2020). https://doi.org/10.1016/j.joule.2020.05.018

[S24] X. Yue, H. Liu, P. Liu, Polymer grafted on carbon nanotubes as a flexible cathode for aqueous zinc ion batteries. Chem. Commun. **55**, 1647 (2019). https://doi.org/10.1039/c8cc10060h

[S25] W. Wang, V.S. Kale, Z. Cao, Y. Lei, S. Kandambeth et al., Molecular engineering of covalent organic framework cathodes for enhanced zinc-ion batteries. Adv. Mater. **33**, 2103617 (2021). https://doi.org/10.1002/adma.202103617

[S26] Z. Song, L. Miao, L. Ruhlmann, Y. Lv, L. Li et al., Proton - conductive supramolecular hydrogen - bonded organic superstructures for high - performance zinc - organic batteries. Angew. Chem. Int. Ed. **62**, e202219136 (2023). https://doi.org/10.1002/anie.202219136

[S27] F. Wan, L. Zhang, X. Wang, S. Bi, Z. Niu et al., An aqueous rechargeable zinc-organic battery with hybrid mechanism. Adv. Funct. Mater. **28**, 1804975 (2018). https://doi.org/10.1002/adfm.201804975

[S28] N. Wang, Z. Guo, Z. Ni, J. Xu, X. Qiu et al., Molecular tailoring of an n/p-type phenothiazine organic scaffold for zinc batteries. Angew. Chem. Int. Ed. **60**, 20826 (2021). https://doi.org/10.1002/anie.202106238

[S29] H. Glatz, E. Lizundia, F. Pacifico, D. Kundu, An organic cathode based dual-ion aqueous zinc battery enabled by a cellulose membrane. ACS Appl. Mater. Interfaces **2**, 1288 (2019). https://doi.org/10.1021/acsaem.8b01851

[S30] C. Zhang, W. Ma, C. Han, L.-W. Luo, A. Daniyar et al., Tailoring the linking patterns of polypyrene cathodes for high-performance aqueous zn dual-ion batteries. Energy Environ. Sci. **14**, 462 (2021). https://doi.org/10.1039/d0ee03356a
